# Deep optimization-guided hybrid neural network for accurate detection and segmentation of white matter hyperintensities in clinical MRI images

**DOI:** 10.1038/s41598-026-41137-7

**Published:** 2026-03-13

**Authors:** Bharathi Panduri, O. Srinivasa Rao

**Affiliations:** Department of Computer Science and Engineering, Jawaharlal Nehru Technological University, Kakinada, India

**Keywords:** Deep Neural Network, Brain MRI Images, White Matter Hyperintensities Segmentation, ResNet50, Inception-v3, Computational biology and bioinformatics, Engineering, Mathematics and computing, Neurology, Neuroscience

## Abstract

White matter hyperintensities (WMHs) are common radiological findings in brain magnetic resonance imaging (MRI) and are strongly associated with neurological disorders such as stroke, dementia, and multiple sclerosis. Accurate detection and segmentation of WMHs are crucial for early diagnosis, disease progression analysis, and treatment planning. However, manual delineation of WMHs is labour-intensive, time-consuming, and prone to inter-observer variability, which limits its practicality in large-scale clinical and research settings. Deep learning has shown promise in automating WMH analysis; however, challenges remain due to heterogeneous lesion sizes, low contrast boundaries, and imaging noise. We propose a Deep Optimization-Guided Hybrid Neural Network (DOGHNN) that combines Inception-v3, ResNet-50, and Practical Swarm Optimization (PSO) for enhanced WMH segmentation. Inception-v3 is employed to capture multi-scale lesion features, enabling the detection of both small punctate and large confluent WMHs. ResNet-50 is integrated to extract deep contextual representations, leveraging residual learning to distinguish true lesions from surrounding tissue and artifacts. Finally, PSO is incorporated as an optimization strategy to iteratively refine fusion weights, segmentation thresholds, and key parameters, minimizing segmentation loss and improving boundary delineation. This hybrid approach ensures both fine-grained lesion sensitivity and robust global feature learning. The DOGHNN framework was evaluated on benchmark WMH MRI datasets with diverse lesion loads and anatomical complexities. Comparative experiments showed superior performance over baseline deep learning models. Quantitative evaluation yielded a maximum precision of 93.2%, recall of 91.5%, dice score 91.1%, and f1-score of 90.5% were achieved by the suggested DOGHNN, and Hausdorff distance of 6.5, confirming its robustness and reliability. By combining multi-scale learning, residual contextual modelling, and optimization-driven refinement, the DOGHNN framework delivers accurate and efficient WMH segmentation. This approach holds strong potential for clinical integration, supporting automated neuroimaging workflows and improving diagnostic decision-making in neurological care.

## Introduction

**Fig. 1:**
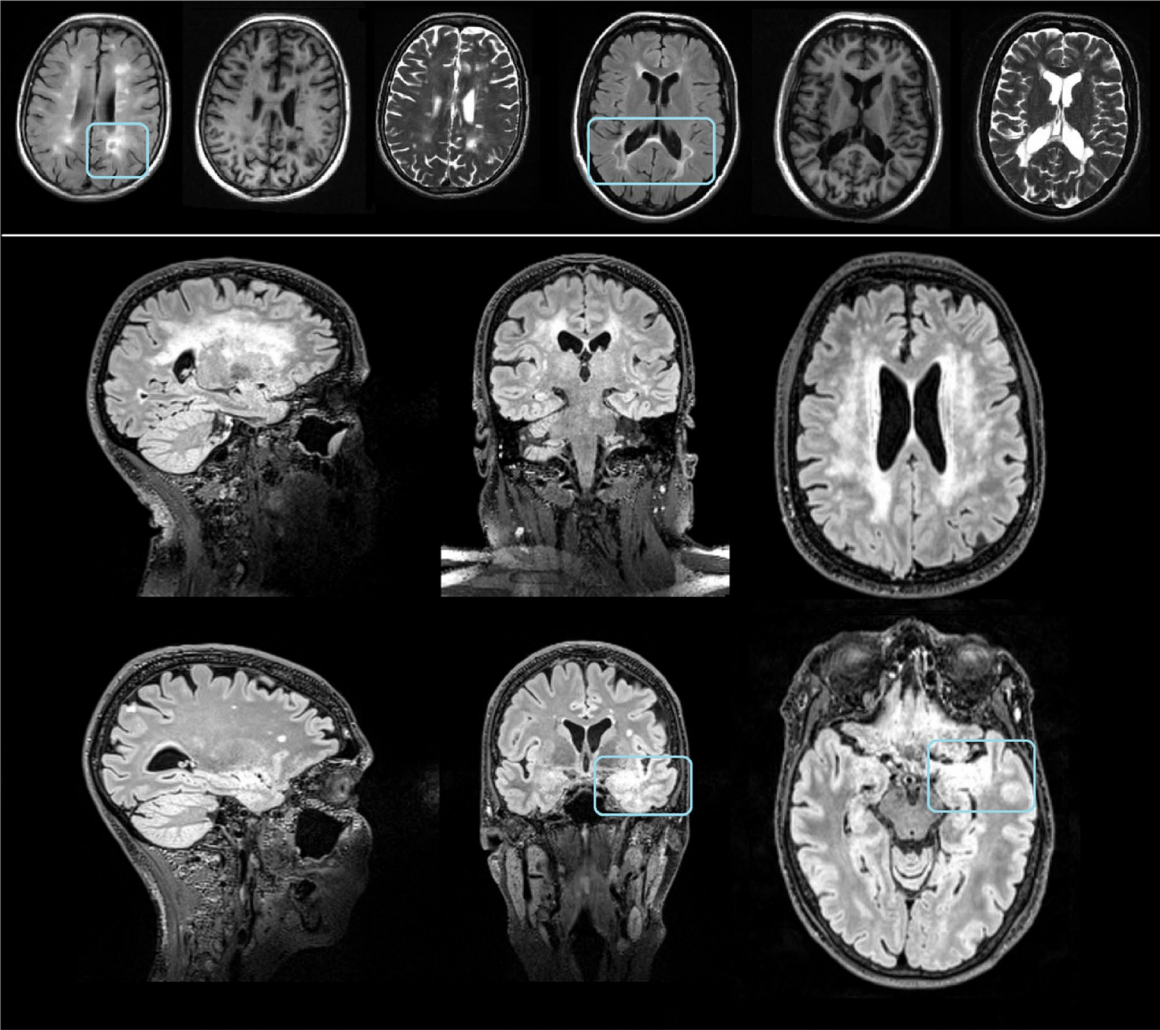
Figure illustrates T2-weighted hyperintensities observed in multiple sclerosis (top row) and isolated cerebral small vessel disease (CSVD) (middle and bottom rows). The top row shows representative axial slices from two patients with white matter hyperintensities (WMH) in T1-w, T2-w, and FLAIR MRI sequences acquired at 1.5T, highlighting pseudo-cavitated hyperintense regions on FLAIR (marked with rectangles). The middle row presents FLAIR images from a 3T MRI scan of a CSVD patient exhibiting extensive WMH, predominantly of vascular origin. The bottom row depicts FLAIR 3T MRI images from another CSVD patient with a low WMH burden of suspected vascular cause, displayed sequentially in sagittal, coronal, and axial planes.

White matter hyperintensities (WMH) refer to areas within the brain that appear as regions of increased signal intensity on T2-weighted (T2-w) or Fluid Attenuated Inversion Recovery (FLAIR) MRI scans. These signal elevations are typically associated with localized changes in brain tissue composition. Although the exact underlying cause of WMH is not clearly defined, they are often observed in a variety of neurological and pathological conditions, including cerebral ischemic lesions, demyelinating disorders, hydrocephalus, traumatic injuries, inflammatory processes, radiation effects, and amyloidosis. In older participants, WMH are common, and in those with cerebrovascular risk factors such diabetes and hypertension, they occur more frequently. On top of that, WMH are prevalent among people who suffer from neurological diseases like stroke, Parkinson’s, moderate cognitive impairment, Alzheimer’s, and even main mental disorders like schizophrenia spectrum disorders and mood disorders^3^.While the exact neuropathological, clinical, and cognitive implications of WMH remain unknown, extensive epidemiological research has linked WMH to deficits in several areas of cognitive function, such as frontal executive functions, explicit memory, and psychomotor speed^4^. Unexpectedly, a meta-analysis and comprehensive review found that WMH is linked to a higher risk of stroke, dementia, and death, suggesting that it could be a significant predictor of future illness^5^. In addition, WMH are common in preclinical stages of dementia (such mild cognitive impairment) and their presence may raise the risk of developing dementia when mild cognitive impairment has already progressed. Their dispersed spatial distribution and inherent unpredictability make their quantification and localization a formidable obstacle to their investigation^6^. There are two methods for analysing WMH in MRI brain scans: quantitative volumetric studies and semi-quantitative rating systems. A number of scales with notably diverse morphological or anatomical definitions have been suggested and utilized in the literature, and their calculation forms the backbone of the semi-quantitative approach^7^. Figure 1, represents T2 hyperintensities in MS (top) and CSVD with high (middle) and low (bottom) WMH burden.

WMH visual rating scales are appealing for large-scale epidemiological studies due to their widespread use in clinical and research settings, ease of use across scanners, and relative speed of execution. Regrettably, they are subject to a variety of restrictions. Indeed, the limited range of values for category evaluations limits the strength of linkage. The interpretive subjectivity of qualitative scales further reduces their reliability and consistency when used in longitudinal investigations^8^. However, numerical approaches to quantifying WMH severity have been proposed by a number of recent studies that employ computer-based methodologies to provide volumetric measurements of WMH burden. These approaches range from completely automated WMH identification to those that require manual outlining. In region-of-interest (ROI) procedures, which involve manual outlining techniques, the tracer examines the scan using visualization software and then sketches WMH areas by hand^9^. Once the section thickness and the number of voxels contained in the traced area have been determined, the volume of each region can easily be determined. A total WMH volume is obtained by adding the values of all sections together^10^. Although accurate, manual outlining processes have a number of drawbacks, including being labor-intensive, time-consuming, subjective, and prone to errors. There is both intra- and inter-observer variability when it comes to manual detection. Improvements in semi-or fully-automated WMH segmentation have been substantial in recent years^11^. Quantitative volumetric data on WMH can be collected using these methods, which are based on computer algorithms that were created to substitute the human eye. The visual WMH evaluations aren’t as objective as these methods, which are free of user bias. Nevertheless, the precision, processing speed, and intricacy of these approaches vary^12^.

Most of these methods use multimodal data, which means they are based on various MRI sequences, such as T2-w, FLAIR, inversion recovery (IR), T1-weighted (T1-w), proton density (PD), and so on. Training images with WMH labels are the foundation of new WMH detection algorithms. These methods include neural classification, k-nearest neighbour, and Markov random field models^13^. Because WMH is so diverse, it can be challenging to choose representative training data, which in turn affects these approaches’ segmentation accuracy. In recent years, deep learning has significantly advanced medical image analysis, particularly in the segmentation and detection of complex brain lesions. WMHs, which play a crucial role in the diagnosis and monitoring of various neurological disorders, present unique challenges due to their heterogeneous size, shape, and intensity variations across patients.

Conventional methods often struggle with accurate delineation, especially in distinguishing subtle lesions from surrounding tissues or artifacts^14^. To address these limitations, we propose a Deep Optimization-Guided Hybrid Neural Network (DOGHNN) that combines the strengths of multiple deep learning models with an optimization strategy for enhanced WMH segmentation. The proposed DOGHNN framework integrates Inception-v3, ResNet-50, and Particle Swarm Optimization (PSO) into a unified architecture. Inception-v3 is employed to capture multi-scale lesion features, enabling precise detection of both small punctate and large confluent WMHs. To further enhance contextual understanding, ResNet-50 is incorporated for extracting deep hierarchical representations through residual learning, allowing the model to differentiate true lesions from noise and surrounding tissue structures. Finally, PSO serves as an optimization layer, iteratively refining fusion weights, segmentation thresholds, and key hyperparameters to minimize segmentation loss and improve boundary delineation. By combining fine-grained lesion sensitivity with robust global feature learning, the DOGHNN framework offers a reliable and automated approach for WMH segmentation.

- To develop a functionally coordinated hybrid architecture (DOGHNN) in which Inception-v3 and ResNet-50 serve distinct and complementary roles—multi-scale lesion sensitivity and deep contextual refinement, respectively—thereby addressing WMH heterogeneity more effectively than conventional parallel or standalone CNN designs.
- To embed Practical Swarm Optimization (PSO) as an adaptive optimization layer that jointly tunes fusion weights, segmentation thresholds, and key parameters, enabling dynamic interaction between network components and overcoming the limitations of fixed or empirically chosen fusion strategies commonly used in prior WMH segmentation studies.
- To demonstrate the superiority of the proposed integration beyond empirical accuracy by linking architectural coordination and optimization-guided refinement to improved boundary delineation, robustness across lesion loads, and consistent performance across multiple clinically relevant evaluation metrics.

The rest of the article is organized like this: The typical WMHs segmentation approaches utilized in the literature and the issues encountered were described in depth in Sect.  2, which served as the research topic for the creation of the suggested method. In Sect.  3, we go over the proposed research technique and dataset. The results and comparisons of the proposed method with existing state-of-the-art methods are presented in Sect.  4, and Sect.  5 summarizes the findings and suggests areas for future research.

## Literature survey

The computing analysis of magnetic resonance imaging (MRI) pictures of the brain encompasses a wider range of applications, including the accurate segmentation and quantification of white matter hyperintensities (WMH). There have been a lot of published studies on WMH detection and segmentation^15^. More study is being done in this area. This paper summarizes previous research on WMH segmentation. In region-of-interest (ROI) procedures, which involve manual outlining techniques, the tracer examines the scan utilizing visualization software’s and then sketches WMH areas by hand^16^. Once the section thickness and the number of voxels contained in the traced area have been determined, the volume of each region will be determined.

Although accurate, manual outlining processes have a number of drawbacks, including being labour-intensive, time-consuming, subjective, and prone to errors. More so, both within and between observers, manual detection is prone to error^17^. There have been tremendous strides in the creation of semi-or fully-automated WMH segmentations in the last several years. These methods enable the quantitative measurement of WMH’s volume by utilizing computer algorithms that were created to substitute the human eye^18^. When contrasted with the visually-based WMH ratings, these methods are more unbiased and impartial^19^. On the other hand, the computing speed, complexity, and accuracy of these algorithms vary. Multimodal data is the backbone of most of these methods, which in turn are based on several MRI sequences, such as T2-w, PD, T1-w, IR, and, most commonly, FLAIR images^20^. Training images with WMH labels is necessary for innovative methods to detect WMH that are based on Markov random field models^21^, k-nearest neighbours^22^, and neural classification^23^. Due to the diverse nature of WMH, it may be challenging to choose representative training data, which in turn affects the segmentation accuracy of these algorithms. As an example, WMH segmentation was carried out by^24^ using basic unsupervised mathematical morpho-logical procedures.

**Table 1:** Some algorithms proposed for solving WMH segmentation.

Sl. No.	References	Procedure
1	Vanderbecq et al.^26^	In order to identify the limits that might include the ideal threshold values, the cluster validity measure was employed to examine the threshold levels’ borders. After that, it used GA on the given boundaries to get the best possible threshold values inside.
2	Ding et al.^27^	Maximizing Kapur’s entropy has led to the suggestion of using a real coded GA with simulated binary crossover to tackle the ISP of medical images. Their effectiveness in addressing the medical image’s ISP was validated by this algorithm in comparison to others.
3	Liu et al.^28^	In order to overcome the ISP, a PSO that is enhanced through collaborative and all-encompassing learning has been created. To protect against early convergence and ease the dimensionality curse, PSO makes use of both comprehensive and cooperative learning.
4	Pitkanen et al.^29^	Adaptive inertia and the adaptive population were used to modify the PSO so that it can tackle the ISP. The adaptive population is employed to avoid becoming stuck in local optima, while adaptive inertia is employed to accelerate PSO’s convergence.
5	Wang et al.^30^	Order of fractions A solution to the picture segmentation issue using the Otsu function has been suggested: Darwinian PSO. To control the pace of convergence, the fractional-derivative was applied with PSO.
6	Hong et al.^31^	To address the ISP using the maximizing otsu technique, WOA and MFA were suggested, but only up to threshold levels of 6.
7	Jeong et al.^32^	As a solution to ISP, this study proposes the Improved FFA (IFFA). IFFA was fine-tuned by utilizing the neighborhood technique to boost convergence and the Cauchy mutation to sidestep local minima.
8	Wei et al.^33^	In order to get the best picture threshold values, the authors of this study suggest using CS to maximize the Tsallis entropy.

Table 1 describes some algorithms proposed for solving WMH segmentation. In addition, a semi-automatic unsupervised segmentation method for WMH was suggested in^25^. Prior to segmenting WMH in acute ischemic stroke disease, they utilized an empirical threshold value and atlas data to identify WMH. But their approach ignored the entire brain in favor of just the hemispheres. A convolutional neural network (CNN) model called U-ResNet^26^ was suggested for the purpose of 2D brain MRI segmentation. It is possible to separate ischemic stroke lesions from WMHs using U-ResNet. When it comes to distinguishing between tiny WMHs and ischemic stroke lesions, U-ResNet falls short, but it excels at segmenting and differentiating larger WMHs. Similarly^34^, suggested a fuzzy inference method that could categorize the WMH using intensity values and anatomical positions from three separate MR images (T2-w, PD, and FLAIR)—all without training samples—in a seamless manner. In addition, the WMH can be automatically or semi-automatically segmented using FLAIR images alone by setting a threshold for the images^35^. Here^36^, used empirical thresholds to divide WMH into smaller pieces before using linear fitting or fuzzy clustering. In a similar vein^37^, estimated the WMH threshold using the mean and standard deviation (SD) of GM, WM, and CSF intensities, and they employed a WM probability map to pinpoint the most probable WM locations.

Recently, a WMH segmentation method was proposed^38^; to determine voxel class probabilities, it employs a modified context-sensitive Gaussian mixture model; to eliminate the usual FLAIR distortions, it employs a false positive correction step. There are limits to all of the quantitative methods that have been discussed here. A great deal of technological resources is needed for complex computer-based segmentation methods, some of which may not be accessible in clinical settings. The capture of all these images is costly and takes a long time to process, therefore multi-spectral techniques aren’t always available in clinical practice^39^. In contrast, FLAIR-based approaches have a tendency to overestimate the WMH load on occasion. This is due to FLAIR’s high intensity appearance in cortical areas, such as the septum pellucidum, and low artifacts in the fourth ventricle, an area where a greater number of false positives are detected^40^.

The absence of automated approaches that have been shown in populations with low prevalence and minor total lesion burden hinders the comprehensive characterisation of WMH in young children. Despite the proliferation of WMH segmentation algorithms and tools, many of which rely on deep learning (DL), this remains the case. Most of the existing methods are optimized for use in older participants or MS patients, who are more likely to have a heavy burden of WMH, as shown by more defined borders and larger confluent lesions^41^. More sophisticated DL-based approaches compared to older, more conventional signal-processing and machine-learning-based approaches show little benefit in these populations^42^. As mentioned earlier, the real benefit of these sophisticated approaches might be more noticeable when it comes to WMH segmentation in individuals with a relatively little lesion load (< 5 ml)^43^.

**Table 2:** Represents state-of-the-art methods for WMH segmentation.

References	Year	Methodology	Dataset	Limitations
Shan et al.^24^	2023	U-Net	BrainWeb	Limited generalization to other datasets
Umapathy et al.^25^	2023	CNN	MICCAI	High computational cost
Philps et al.^11^	2023	DeepLabV3	IBSR	Needs extensive parameter tuning
Gibson et al.^10^	2024	GANs	ADNI	Complexity of integrating GANs
Bawil et al.^2^	2024	PSO + CNN	IBSR	Balancing multiple objectives
Banu et al.^6^	2024	3D CNN	MICCAI BRATS	High memory requirements
Ucar et al.^7^	2025	Transfer learning	ADNI	Limited transferability across domains
Mu and Li^8^	2025	InceptionNet	BrainWeb, IBSR	Real-time constraints
Bawil et al.^9^	2025	Ensemble learning + PSO	IBSR	Complexity of ensemble methods, Effectiveness of PSO tuning
Zhang et al.^14^	2025	Edge U-Net	BrainWeb, IBSR	Limited transferability across domains

The inability to detect little WMH off-plane is a major drawback of databases that use 2D FLAIR acquisition and slice thicknesses between 3 and 5 mm. Even studies that test their strategy in light WMH load individuals using DL-based^44^ or other methods^45^ nevertheless fall into this category. Table 2, represents state-of-the-art methods for WMH segmentation. In high-quality 3D FLAIR scans from healthy young-to-medium-aged adults with very modest WMH load, no study has maximized the segmentation performance to our knowledge. Among the deep learning-based approaches, the U-Net-based architecture^6^ has been by far the most popular and successful. Based on this, the top two winning methods in the MICCAI 2017 WMH segmentation competition^7^ used versions of the U-Net architecture^6^. U-Net is perfect for pixel-by-pixel classification problems in biomedical image segmentation because of its complex design, which allows for successful learning from sparse training picture sources^8^. In contrast to numerous well-publicized neural networks for picture classification—for example, ImageNet with its 1.2 million training images—the U-Net model trained with just 30 datasets of electron microscopy images effectively segmented neuronal structure. In a fully convolutional network, the “u-shape” design is the product of a sequence of contracting layers and up-sampling operators that are symmetrical to the contracting path. Linking the feature maps of the contracting path to the expanding path using skip connections allows the model to learn fine-grained detail while preserving spatial information and taking into account the full input image’s spatial context.

### Problem statement

Accurate detection and segmentation of White Matter Hyperintensities (WMHs) in brain MRI is crucial for early diagnosis, disease monitoring, and treatment planning in neurological disorders such as stroke, dementia, and multiple sclerosis. However, existing deep learning–based approaches often face significant challenges in precisely delineating WMH boundaries due to their heterogeneous appearance, small size, variable shape, and low contrast against surrounding tissues. These challenges are further exacerbated by imaging artifacts, noise, partial volume effects, and intensity inhomogeneities, which can obscure or distort lesion boundaries. Current methods also lack robust optimization strategies to refine segmentation thresholds, fusion weights, and other key parameters, leading to reduced accuracy and generalizability across diverse datasets. Hence, there is a pressing need for a novel, optimization-guided framework that integrates advanced deep neural architectures with metaheuristic optimization techniques to ensure fine-grained WMH detection, robustness against artifacts, computational efficiency for near-real-time clinical use, and interpretable outputs that provide reliable insights for clinical decision-making.

## Methodology

In this study, we introduce a Deep Optimization-Guided Hybrid Neural Network (DOGHNN) as an innovative framework by combining Inception-v3 and ResNet-50 for augmenting WMH detection and segmentation in brain MRI images. Unlike conventional approaches that depend solely on the discriminative power of neural networks, DOGHNN integrates advanced optimization strategies into the learning process. This integration enhances the network’s ability to capture subtle WMH features, refine boundary delineation, and improve segmentation accuracy even in the presence of artifacts and intensity inhomogeneities. In this methodology section, we present a comprehensive overview of the proposed framework, covering its architectural design, training pipeline, and key components. Specifically, we elaborate on the seamless incorporation of PSO within the deep learning framework. PSO plays a pivotal role in refining fusion weights, segmentation thresholds, and critical parameters, thereby guiding the network toward optimal solutions. This synergy between deep neural architectures and optimization techniques forms the foundation of DOGHNN, enabling robust, fine-grained, and clinically reliable WMH segmentation.

### Dataset description

To validate the robustness and effectiveness of the proposed methodology, experiments were conducted using the WMH Segmentation Challenge 2017 dataset^12,13^. The dataset provided a training dataset and a test dataset consisting of 900 subjects in total. Tables 3 and 4^44^ summarize the demographic features of the subjects in each dataset. This dataset (https://wmh.isi.uu.nl/data/, DOI:10.1002/hbm.25695, https://grand-challenge.org/challenges/)^1,21^ (DOI: 10.3389/fnagi.2022.915009, DOI: 10.1109/TMI.2019.2905770) was partitioned into the following three subsets: IDS 1, IDS 2, and a silver standard dataset. IDS 1 consists of 276 subjects acquired using a 3T MRI scanner, while IDS 2 contains 74 subjects acquired using a 1.5T MRI scanner. These two subsets were used for model training and validation. During training, a fixed portion of the combined IDS 1 and IDS 2 datasets was reserved for validation to monitor convergence, perform model selection, and prevent overfitting. The silver standard dataset, comprising 550 subjects with scans obtained from both 3T and 1.5T MRI scanners, was used exclusively for independent testing. This test set was not accessed at any stage during model training, validation, or parameter optimization, thereby ensuring an unbiased and fair evaluation of model performance.

**Table 3:** MRI acquisition protocols.

Parameter (unit)	UIH uMR 780	GE Signa HDxt	GE discovery MR750	GE Signa excite	GE Signa HDxt	GE Signa creator	GE Brivo MR355
Magnetic field strength (T)	3.0	3.0	3.0	1.5	1.5	1.5	1.5
TR/TE/TI (ms)	8000/106/2425	8002/170/2100	8400/115/2200	8602/120/2100	8802/122/2200	8400/102/2100	8000/131/2100
Pixel spacing (mm)	0.5047 * 0. 5047	0.4688 * 0.4688	0.4688 * 0.4688	0.4688 * 0.4688	0.4688 * 0.4688	0.4688 * 0.4688	0.4688 * 0.4688
Inter-slice gap (mm)	1.5	2	2	2	2	2	2
FLAIR							
Slice thickness (mm)	5.5	6	6	6	6	6	6
Matrix	456 * 396	512 * 512	512 * 512	512 * 512	512 * 512	512 * 512	512 * 512
No. of slices	18	16	16	16	16	16	16
TR/TE/TI (ms)	1800/11/790	1785/24/720	1750/24/780	2120/12/700	2180/11/720	2430/19/750	2311/20/750
Pixel spacing (mm)	0.5047 * 0. 5047	0.4688 * 0.4688	0.4688 * 0.4688	0.4688 * 0.4688	0.4688 * 0.4688	0.4688 * 0.4688	0.4688 * 0.4688
Inter-slice gap (mm)	1.5	2	2	2	2	2	2
T1-weighted							
Slice thickness (mm)	5.5	6	6	6	6	6	6
Matrix	456 * 396	512 * 512	512 * 512	512 * 512	512 * 512	512 * 512	512 * 512
No. of slices	18	16	16	16	16	16	16
TR/TE (ms)	4000/93	3800/120	3800/105	4220/103	3280/103	3750/113	3820/116
Pixel spacing (mm)	0.5047 * 0. 5047	0.4688 * 0.4688	0.4688 * 0.4688	0.4688 * 0.4688	0.4688 * 0.4688	0.4688 * 0.4688	0.4688 * 0.4688
Inter-slice gap (mm)	1.5	2	2	2	2	2	2
T2-weighted							
Slice thickness (mm)	5.5	6	6	6	6	6	6
Matrix	456 * 396	512 * 512	512 * 512	512 * 512	512 * 512	512 * 512	512 * 512
No. of slices	18	16	16	16	16	16	16

**Table 4:** Subject distribution across imaging devices.

Scanner	Number	Total
3TGE Signa HDxt	350	900
3TGE Discovery MR750		
3TUIH uMR 780		
1.5TGE Signa Excite	550	
1.5TGE Signa HDxt		
1.5TGE Signa Creator		
1.5TGE Brivo MR355		

In order to train and evaluate our technique, we added some preprocessing to these data. To start, we improved the accuracy by reducing false positives by removing non-brain tissue using Robust Brain Extraction (ROBEX). ROBEX (Robust Brain Extraction) is an automated skull-stripping method that accurately separates brain tissue from non-brain structures in MRI images. It combines a learning-based model with a deformable surface approach to estimate and refine the brain boundary, making it robust to noise, intensity variations, and anatomical differences. By effectively removing non-brain tissue, ROBEX reduces false positives and improves the reliability of subsequent WMH segmentation. The intensity values were then normalized so that they fit within the range of the training data. Using intensities ranging from the 2nd to the 95th percentiles, we calculated the means and standard deviations for every brain image. After that, we adjusted the z-score so that the brain’s intensity was consistent throughout all of the images. Lastly, in order to standardize the input data for the network, the axial slices of every 3D image were either cut or padded to a size of 200 × 200. We trained our suggested model using 2D slices. Table 3 details the techniques for MRI acquisition, while Table 4 shows the distribution of subjects among imaging devices.

### Data pre-processing

In MRI brain imaging, the intensity of voxels reflects tissue characteristics, and preprocessing aims to enhance contrast, suppress noise, and normalize intensities for reliable segmentation. Let $\:I(x,y,z)$ denote the intensity of a voxel at coordinates ($\:x,y,z$) in a 3D MRI volume. Probability density function (PDF) is estimated as:

1 $$\:{p}_{C}\left(I\right)=\frac{1}{{N}_{C}}\sum\:_{i=1}^{{N}_{C}}\:\delta\:\left(I-{I}_{C}^{i}\right)$$

where $\:{I}_{C}^{i}$ is the intensity of the $\:{i}^{th}$ voxel of class $\:C,{N}_{C}$ is the total number of sampled voxels, and $\:\delta\:(\cdot\:)$ is the Dirac delta function༎This PDF models the likelihood of observing a specific intensity in a tissue class. The mean intensity of each class, representing its central tendency, is computed as:

2 $$\:{\mu\:}_{C}=\frac{1}{{N}_{C}}\sum\:_{i=1}^{{N}_{C}}\:{I}_{C}^{i}$$

while the standard deviation, capturing intensity variability, is calculated by:

3 $$\:{\sigma\:}_{C}=\sqrt{\frac{1}{{N}_{C}}\sum\:_{i=1}^{{N}_{C}}\:\:{\left({I}_{C}^{i}-{\mu\:}_{C}\right)}^{2}}$$

The maximum intensity within the brain tissue class, denoted $\:{N}_{\mathrm{max\:}}^{B}$, is given by:

4 $$\:{N}_{\mathrm{m}\mathrm{a}\mathrm{x}}^{B}=\underset{i\in\:\mathrm{\:hrain\:voxels\:}}{\mathrm{m}\mathrm{a}\mathrm{x}}\:{I}_{B}^{i}$$

which serves as a reference for scaling intensity ranges. For a new image, we identify the intensity corresponding to the highest probability in the Probability Density Function (PDF), $\:{N}_{\mathrm{PDF\:}}$, assuming healthy brain tissue dominates the volume. The WMH and vessel mean intensities are then adjusted relative to this maximum:

5 $$\:{\mu\:}_{T}^{\mathrm{n}\mathrm{e}\mathrm{w}}={N}_{\mathrm{P}\mathrm{D}\mathrm{F}}-{N}_{\mathrm{m}\mathrm{a}\mathrm{x}}^{B}+{\mu\:}_{T},\:{\mu\:}_{V}^{\mathrm{n}\mathrm{e}\mathrm{w}}={N}_{\mathrm{P}\mathrm{D}\mathrm{F}}-{N}_{\mathrm{m}\mathrm{a}\mathrm{x}}^{B}+{\mu\:}_{V}$$

To enhance contrast, the lower and upper bounds for piecewise linear transformation are defined as:

6 $$\:{P}_{\mathrm{m}\mathrm{i}\mathrm{n}}={\mu\:}_{T}^{\mathrm{new\:}}-3{\sigma\:}_{T}$$

7 $$\:{P}_{\mathrm{m}\mathrm{a}\mathrm{x}}={\mu\:}_{V}^{\mathrm{new\:}}+3{\sigma\:}_{V}$$

The function that transforms the voxel intensity values, $\:I(x,y,z)$, to a new value, $\:{I}^{{\prime\:}}(x,y,z)$, within the standard range of 0 to 255 is expressed as:

8 $$\:{I}^{{\prime\:}}(x,y,z)=\left\{\begin{array}{ll}\left(\frac{I(x,y,z)-{P}_{\mathrm{m}\mathrm{i}\mathrm{n}}}{{P}_{\mathrm{m}\mathrm{a}\mathrm{x}}-{P}_{\mathrm{m}\mathrm{i}\mathrm{n}}}\right)\times\:254+1,&\:\mathrm{\:if\:}{P}_{\mathrm{m}\mathrm{i}\mathrm{n}}\le\:I(x,y,z)\le\:{P}_{\mathrm{m}\mathrm{a}\mathrm{x}}\\\:0,&\:\mathrm{\:otherwise\:}\end{array}\right.$$

This function essentially stretches the contrast of the intensity values that fall between $\:{P}_{\mathrm{min\:}}$ and $\:{P}_{\mathrm{max\:}}$ to fit the full range of 1 to 255, while setting all other intensity values to 0 This function rescales the tissue specific intensity ranges while suppressing background noise. To reduce impulsive “salt-and-pepper” noise while preserving edge, a median filter is applied:

9 $$\:{I}^{{\prime\:}{\prime\:}}(i,j,k)=\mathrm{m}\mathrm{e}\mathrm{d}\mathrm{i}\mathrm{a}\mathrm{n}\left\{{I}^{{\prime\:}}(o,b,c)\mid\:(o,b,c)\in\:{G}_{i,j,k}\right\}$$

where $\:{G}_{i,j,k}$ is a local neighbourhood window around voxel ($\:i,j,k$), and the median operator selects the middle intensity value within the window. Optionally, gaussian smoothing may be employed tr $\:\downarrow\:$ ther reduce high-frequency noise:

10 $$\:{I}_{s}(x,y,z)=\sum\:_{i=-k}^{k}\:\sum\:_{j=-k}^{k}\:\sum\:_{k=-k}^{k}\:G(i,j,k;\sigma\:){I}^{{\prime\:}{\prime\:}}(x-i,y-j,z-k),$$

with the Gaussian kernel defined as:

11 $$\:G(i,j,k;\sigma\:)=\frac{1}{{\left(2\pi\:{\sigma\:}^{2}\right)}^{3/2}}\mathrm{e}\mathrm{x}\mathrm{p}\left(-\frac{{i}^{2}+{j}^{2}+{k}^{2}}{2{\sigma\:}^{2}}\right)$$

where $\:\sigma\:$ determines the smoothing strength. To normalize intensity values across patients, Z-score normalization is applied:

12 $$\:{I}_{n}(x,y,z)=\frac{{I}_{s}(x,y,z)-{\mu\:}_{B}}{{\sigma\:}_{B}}$$

followed by min-max normalization to scale the intensities between 0 and 1:

13 $$\:{I}_{m}(x,y,z)=\frac{{I}_{n}(x,y,z)-\mathrm{m}\mathrm{i}\mathrm{n}\left({I}_{n}\right)}{\mathrm{m}\mathrm{a}\mathrm{x}\left({I}_{n}\right)-\mathrm{m}\mathrm{i}\mathrm{n}\left({I}_{n}\right)}$$

Candidate WMH are identified using an adaptive threshold based on the normalized brain tissue distribution:

14 $$\:{T}_{\mathrm{W}\mathrm{M}\mathrm{H}}={\mu\:}_{B}+k{\sigma\:}_{B},\:{I}_{\mathrm{W}\mathrm{M}\mathrm{H}}(x,y,z)=\left\{\begin{array}{ll}1,&\:{I}_{m}(x,y,z)\ge\:{T}_{\mathrm{W}\mathrm{M}\mathrm{H}}\\\:0,&\:\mathrm{\:otherwise\:}\end{array}\right.$$

where $\:k$ is a scaling factor controlling sensitivity. Morphological opening removes small noise clusters while preserving WMH structures:

15 $$\:{I}_{\mathrm{m}\mathrm{o}\mathrm{r}\mathrm{p}\mathrm{h}}=\left({I}_{\mathrm{W}\mathrm{M}\mathrm{H}}\ominus\:S\right)\oplus\:S$$

with $\:S$ as a structuring element, $\:\ominus\:$ denoting erosion, and $\:\oplus\:$ denoting dilation. The total segmented WMH volume is then computed as:

16 $$\:{V}_{\mathrm{W}\mathrm{M}\mathrm{H}}=\sum\:_{x,y,z}\:{I}_{\mathrm{morph\:}}(x,y,z)\cdot\:{v}_{\mathrm{voxel\:}},$$

where $\:{v}_{\mathrm{voxel\:}}$ is the physical voxel volume. Finally, for input into the deep hybrid neural network, the processed image may be weighted by tissue classes:

17 $$\:{I}_{\mathrm{input\:}}(x,y,z)={w}_{B}{I}_{m}(x,y,z)+{w}_{T}{I}_{\mathrm{WMH}\text{}}(x,y,z)+{w}_{V}{I}_{\mathrm{vessel\:}}(x,y,z)$$

where $\:{w}_{B},{w}_{T},{w}_{V}$ are weights reflecting the relative importance of each tissue type. This preprocessing pipeline ensures that intensity distributions are standardized, noise is suppressed, and key structures like white matter hyperintensities are preserved, providing optimal input for deep learning segmentation.

### Proposed model

The DOGHNN method combines ResNet50 for feature extraction, Inception-v3 for contextual understanding, and PSO for fine-tuning. After preprocessing, ResNet50 extracts features, while Inspection-v3 refines contextual understanding. PSO guides training for optimized performance. Trained DOGHNN models accurately detect WMH boundaries in MRI images. Evaluation metrics validate its effectiveness, ensuring robustness. Iterative refinement leads to deployment for clinical use, aiding radiologists in accurate diagnosis and treatment planning.

#### ResNet50 model

**Fig. 2:**
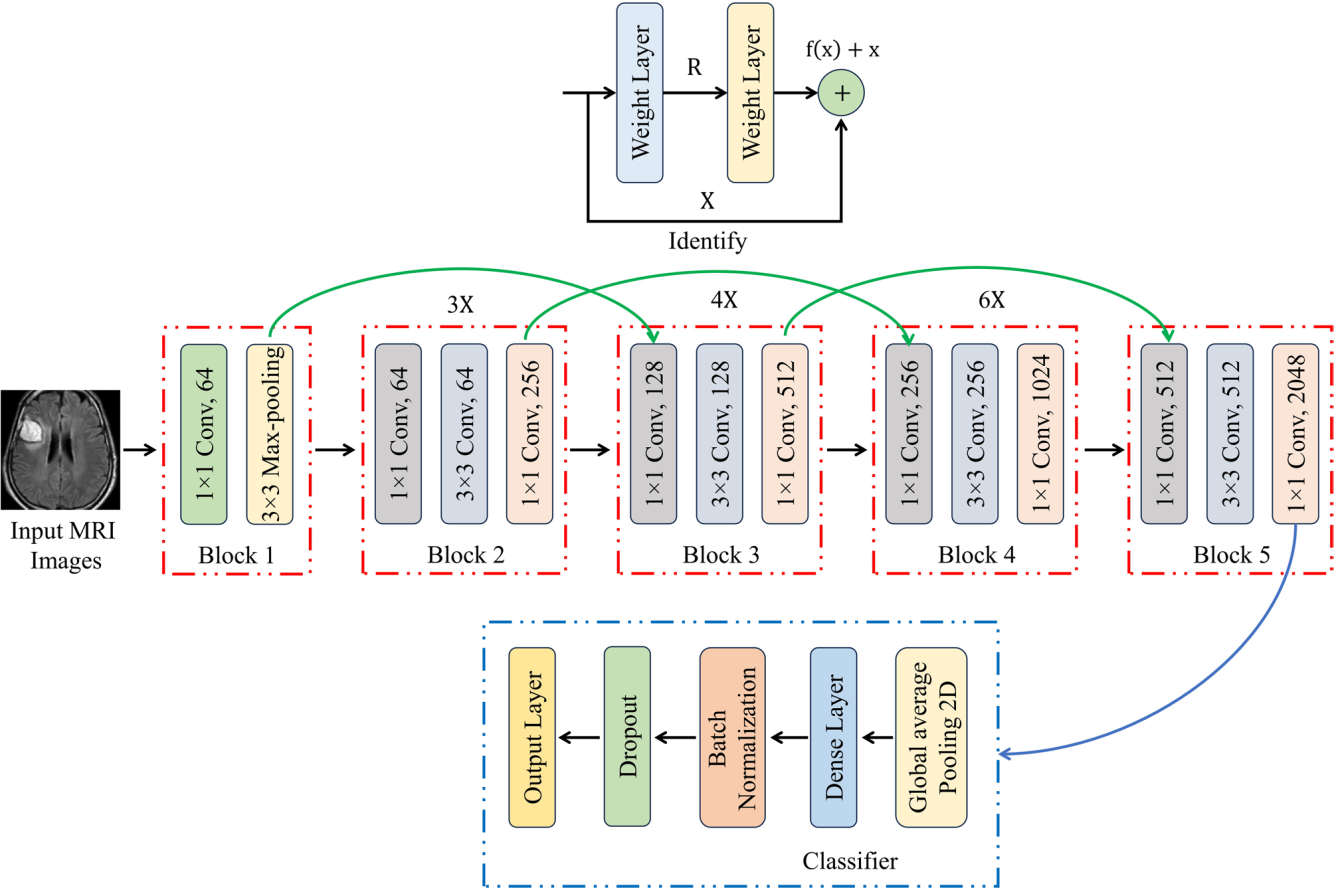
Architecture of the ResNet50V2 model.

ResNet50 model fine-tuned with CNN has become a standard technique for detecting brain WMHs (Brain Tissue) using MRI scans. Originally trained on the extensive ImageNet dataset for general image recognition tasks, ResNet50 consists of various layers including convolutional, pooling, and fully connected layers. In the context of brain WMH detection, ResNet50 serves as a feature extractor, with its lower layers learning fundamental image features beneficial for WMH identification. The common practice involves substituting the final layers of ResNet50 with a tailored set of fully connected layers designed for brain WMH detection and categorization. Following this, the complete model undergoes fine-tuning on a fresh dataset of MRI scans, with weights updated across all layers through backpropagation and stochastic gradient descent. The input data consists of brain MRI scans, often pre-processed to amplify the contrast between WMHs and surrounding tissue. The fine-tuned model generates voxel-wise probability maps indicating the likelihood of WMH presence, which are subsequently thresholder to produce the final WMH segmentation mask. Throughout the training phase, the refined ResNet50 model with CNN learns to identify distinctive features in brain MRI scans and classify them as either WMH or healthy tissue. Utilizing the pre-trained ResNet50 model establishes a strong initial feature set for WMH detection, while fine-tuning on a new MRI dataset ensures adaptation to the specific detection task. This methodology has shown significant accuracy in detecting and classifying brain WMHs using MRI scans. The depicted framework for brain WMH detection employing CNN-ResNet50 is illustrated in Fig. 2 below. The proposed classification framework comprises the following elements:

Image extraction: Histogram Equalization on Fuzzy-based Improved Particle Swarm Optimization (FIPSO) is a histogram approach that is detailed in reference^26^ and is used to enhance the contrast in the MRI images that are obtained from scan sequences. This method eliminates blurriness by using the Non-subsampled Contourlet Transform and uses Gaussian functions to spread pixel intensity among surrounding pixels. It also improves picture details by smoothing. To determine which pixels are bright and which are dark, local maxima are calculated. Furthermore, all local maxima intervals are given relevance in the Takagi-Sugeno Kang model^43^, which fuzzifies the smoothed images.

Best quality patch selection and Fusion: To enhance the resolution of magnetic resonance imaging (MRI) pictures, a local patch-based super resolution (SR) method is used rather than a global SR methodology. This technique involves slicing the low-resolution (LR) picture into smaller, square-shaped pieces. During the super-resolution process, possible problems with local deformation can be handled by using local patches. The magnetic resonance imaging (MRI) pictures are divided into 8 × 8 sections. These patches are geographically clustered because they originate from the same spatial region yet come from distinct photos. A first-order derivative patch, created with the Sobel filter, is applied to each of these images to improve them. Subsequently, the LR images obtained from the selected enhanced patches are combined using the Discrete Cosine Transform (DCT) in conjunction with the ResNet50-based fusion technique.

Consider an image $\:F=\left\{{f}_{(i,j)}\right\}$ where $\:i=\mathrm{0,1},2,\dots\:,N-1$ and $\:j=\mathrm{0,1},2,\dots\:,M-1$. The image is partitioned into $\:P$ non-overlapping blocks, each of size $\:8\times\:8$ pixels. Let $\:{B}_{n}=\left\{{b}_{(n,k,l)}\right\}$ represent the $\:{n}^{\mathrm{th\:}}$ block, where $\:n=\mathrm{0,1},2,\dots\:,P-1,k=\mathrm{0,1},\dots\:,7$, and $\:l=\mathrm{0,1},\dots\:,7$. The Discrete Cosine Transform (DCT) of each block $\:{B}_{n}$ yields the transformed coefficients $\:{Y}_{n}=\left\{{y}_{(n,k,l)}\right\}$. The complete DCT representation of the image $\:F$ is denoted as $\:D=\left\{{D}_{0},{D}_{1},{D}_{2},\dots\:,{D}_{P-1}\right\}$. Similarly, the DCT of the $\:{R}^{\mathrm{th\:}}$ input image is expressed as $\:{D}^{R}=\left\{{D}_{0}^{R},{D}_{1}^{R},{D}_{2}^{R},\dots\:,{D}_{P-1}^{R}\right\}$.

**Fig. 3:**
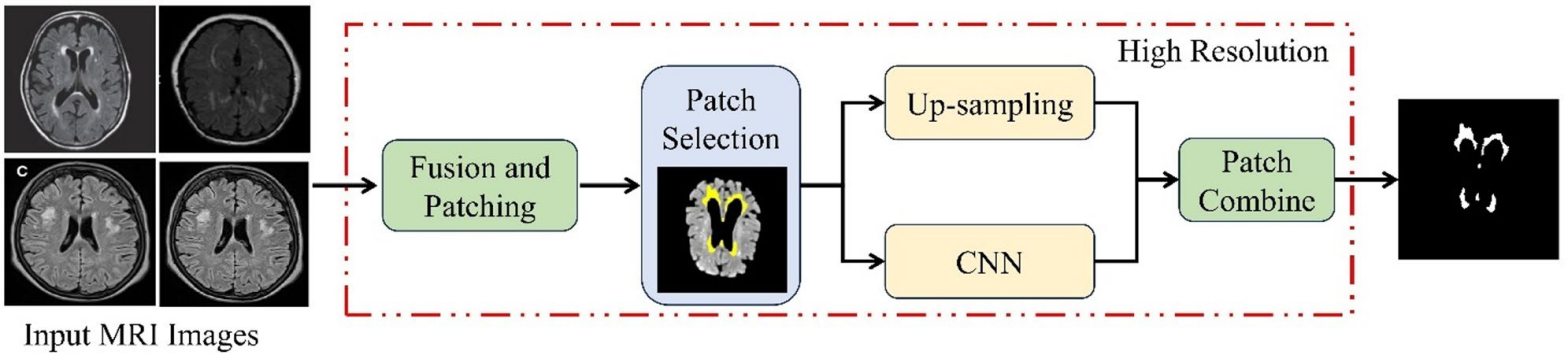
ResNet50 brain WMH classification framework.

The DCT of the final fused image is represented by $\:{D}^{F}=\left\{{D}_{0}^{F},{D}_{1}^{F},{D}_{2}^{F},\dots\:,{D}_{P-1}^{F}\right\}$, which is obtained by merging the corresponding DCT coefficients from images $\:A$ and $\:B$. In this process, let $\:{P}_{n}^{A}$ and $\:{P}_{n}^{B}$ denote the $\:{n}^{\mathrm{th\:}}$ blocks of images $\:A$ and $\:B$, respectively. The algorithm begins by computing the DCT coefficients for each corresponding block from both images.

18 $$\:{D}_{n}^{A}=DCT\:\left({P}_{n}^{A}\right)\:\mathrm{a}\mathrm{n}\mathrm{d}\:{D}_{n}^{B}=DCT\:\left({P}_{n}^{B}\right)$$

The two-dimensional Discrete Cosine Transform (DCT) formula is provided below. This operation is applied to an N × N square matrix of pixel values, resulting in an N × N square matrix of frequency coefficients. The DCT^27^ is calculated as below:

19 $$\:\begin{array}{c}F(u,v)=\frac{2}{N}c\left(u\right)c\left(v\right)\sum\:_{y=0}^{N-1}\:\sum\:_{x=0}^{N-1}\:f(x,y)cos\left[\frac{(2x+1)u\pi\:}{2N}\right]\\\:\times\:cos\left[\frac{(2y+1)v\pi\:}{2N}\right]\end{array}$$

where u, v = 0, 1, …, *N* − 1, with

20 $$\:c\left(u\right)=\left\{\begin{array}{c}\frac{1}{\sqrt{N}},\hspace{0.25em}\hspace{0.25em}\hspace{0.25em}\hspace{0.25em}\text{}\mathrm{i}\mathrm{f}\text{}u=0\\\:\frac{\sqrt{2}}{\sqrt{N}}1,\hspace{0.25em}\hspace{0.25em}\hspace{0.25em}\hspace{0.25em}\text{}\mathrm{i}\mathrm{f}\text{}u\ne\:0\end{array}\right.$$

Here, $\:\mathrm{c}\left(\mathrm{u}\right)=\mathrm{c}\left(\mathrm{v}\right)\:\mathrm{a}\mathrm{s}\:\mathrm{N}\times\:\mathrm{N}$ square matrix of pixel values is used.

- Extract the AC and DC coefficients from both blocks and set the DC coefficients to zero.
  21 $$\:{\mathrm{D}}_{\mathrm{n}\left(0\right)}^{\mathrm{A}}=0\mathrm{\:and\:}{\mathrm{D}}_{\mathrm{n}\left(0\right)}^{\mathrm{B}}=0$$
- Compute the squares of all AC components within the coefficients of blocks extracted from images A and B:
  22 $$\:{\left[{D}_{n(1:63)}^{A}\right]}^{2}\mathrm{\:and\:}{\left[{D}_{n(1:63)}^{B}\right]}^{2}$$
- Add coefficients from 1 to 31 of blocks from images A and B.
  23 $$\:{\mathrm{L}}_{\mathrm{n}}^{\mathrm{A}}={\mathrm{D}}_{\mathrm{n}(1:31)}^{\mathrm{A}}\:\mathrm{\:and\:}\:{\mathrm{L}}_{\mathrm{n}}^{\mathrm{B}}={\mathrm{D}}_{\mathrm{n}(1:31)}^{\mathrm{B}}$$
- Add coefficients from 32 to 63 of blocks of images A and B:
  24 $$\:{\mathrm{H}}_{\mathrm{n}}^{\mathrm{A}}={\mathrm{D}}_{\mathrm{n}(32:63)}^{\mathrm{A}}\mathrm{\:and\:}{\mathrm{H}}_{\mathrm{n}}^{\mathrm{B}}={\mathrm{D}}_{\mathrm{n}(32:63)}^{\mathrm{B}}$$
- Determine the quality of each block using this equation:
  25 $$\:B{Q}_{n}^{A}=\frac{{H}_{n}^{A}}{{L}_{n}^{A}}\mathrm{\:and\:}B{Q}_{n}^{B}=\frac{{H}_{n}^{B}}{{L}_{n}^{A}}$$
- Assess the quality of each block to determine which one is chosen for constructing the fused image based on this criterion.
  26 $$\:B{Q}_{n}^{A}>B{Q}_{n}^{B},\:\mathrm{t}\mathrm{h}\mathrm{e}\mathrm{n}\:{D}_{n}^{F}={D}_{n}^{A}$$
  Else.
  27 $$\:{D}_{n}^{F}={D}_{n}^{B}$$
- Implement the consistency verification procedure^28^ to prevent incorrect block selections. This verification method employs a 3 × 3 neighbourhood window.
- Repeat the aforementioned steps for all P blocks to merge DCT coefficients from multiple images into a unified DCT representation of the image.

Utilize the inverse Discrete Cosine Transform (IDCT) to merge the DCT coefficients and reconstruct the fused image. The IDCT is derived from the following expression:

28 $$\:\begin{array}{c}f(x,y)=\frac{2}{N}\sum\:_{v=0}^{N-1}\:\sum\:_{u=0}^{N-1}\:c\left(u\right)\left(v\right)F(u,v)cos\left[\frac{(2x+1)u\pi\:}{2N}\right]\\\:\times\:cos\left[\frac{(2y+1)v\pi\:}{2N}\right],\end{array}$$

where x, y = 0, 1, …, *N* − 1.

##### Patch selection and super-resolution

In order to improve the fused image’s resolution, the local patch-based super-resolution (SR) method is used. The complete image is segmented into 10 × 10 patches using this technique. The content of each patch is checked by determining its variance; if the variance is greater than a threshold that has been previously set, the patch is sent to the CNN block for SR. In contrast, the patch is sent to the up-sampling block if the variance is less than the threshold that has been previously specified. Using the CNN block to process the sparsest patch could potentially increase processing time. In order to speed up the SR process and solve this issue, the patch with the least information is up-sampled. Figure 3 provides an overview of the ResNet50 brain WMH categorization framework. The following procedures describe the fused image patch’s super-resolution in detail.

Block I: A representation block describes this particular building block. Patches are obtained and represented utilizing a collection of pre-trained bases as part of the image reconstruction process. The patch is convolved with a set of filters as part of this technique. A set of Discrete Cosine Transform bases that have been pre-trained are used to extract and describe overlapping image patches. The up-sampled image based on Bicubic interpolation is denoted by X, whereas the low-resolution (LR) image is represented by x. The next step is to use image F(X) and X as inputs to recreate the WMH so that it looks like the reference HR image X. This strategy is based on the following reasoning.

29 $$\:{F}_{1}\left(x\right)=\mathrm{m}\mathrm{a}\mathrm{x}(0,{s}_{1}\times\:\mathrm{X}+{V}_{1})$$

Where, $\:{V}_{1}-{n}_{1}$ dimensional vector, in which element is associated with a filter, $\:{\mathrm{S}}_{1}$-Convolution filter having a size of $\:\mathrm{m}\times\:{\mathrm{f}}_{1}\times\:{\mathrm{f}}_{1}\times\:{\mathrm{n}}_{1}$, $\:{\mathrm{n}}_{1}$-Number of filters, $\:{\mathrm{f}}_{1}$-Spatial size of the filter, m-Number of channels. The output has $\:{\mathrm{n}}_{1}$ feature maps.

Block II: Block for non-linear mapping. To create $\:{n}_{2}$-dimensional feature vectors, $\:{n}_{1}$-dimensional feature vectors are transformed by.

30 $$\:{\mathrm{F}}_{2}\left(X\right)=\mathrm{m}\mathrm{a}\mathrm{x}\left(0,{S}_{2}\mathrm{*}{\text{}\mathrm{F}}_{1}\left(Y\right)+{V}_{2}\right)$$

where, $\:{\mathrm{n}}_{2}$-number of feature maps, $\:{V}_{2}-{\mathrm{n}}_{2}$ dimensional vector, and $\:{S}_{2}-\mathrm{s}\mathrm{i}\mathrm{z}\mathrm{e}\:{\mathrm{n}}_{1}\times\:1\times\:1\times\:{\mathrm{n}}_{2}$.

Block III: Reconstruction Block. The predicted HR patches are averaged to produce the final HR image given by.

31 $$\:\mathrm{F}\left(X\right)={S}_{3}\mathrm{*}{\text{}\mathrm{F}}_{2}\left(X\right)+{V}_{3}$$

where,

$$V_{3} - {\mathrm{l}} - {\mathrm{dimensional}}\:{\mathrm{vector}},\:S_{3} - {\mathrm{size}}\:{\mathrm{n}}_{2} \times \:{\mathrm{f}}_{3} \times \:{\mathrm{f}}_{3} \times \:{\mathrm{k}}.$$

In order to learn the end-to-end mapping function F, the network parameter $\:K = ({\mathrm{W}}_{1} ,{\mathrm{W}}_{2} ,{\mathrm{W}}_{3} ,V_{1} ,V_{2} ,V_{3} ),$ must be determined during the training phase. Get these network parameters by finding the loss function that minimizes the difference between the original high-resolution image X and the reconstructed images $\:\mathrm{F}(X,K)$

32 $$\:{\mathrm{L}}\left( K \right) = \frac{1}{{\mathrm{n}}}\sum\limits_{{{\mathrm{i}} = 1}}^{{_{{}}^{{\mathrm{n}}} }} {\left\| {{\mathrm{F}}\left( {{\mathrm{x}}_{{\mathrm{i}}} ,K} \right) - {\mathrm{x}}_{{\mathrm{i}}} } \right\|^{2} }$$

where, $\:{x}_{\mathrm{i}}$-LR image, and $\:{X}_{\mathrm{i}}$-Corresponding HR image.

The loss is minimized by PSO with the standard back-propagation method.

#### Inception v3 model

This study used pre-trained inception-v3 models based on CNNs to improve performance and distinguish between disease-affected and healthy images. The main model utilizes data from previously trained ResNet50 models to effectively segment and analyze brain WMH images. The CNN model, which was trained to function as a WMH identification method, has retained new field images. Improved the inception-v3 model were large kernel filters with 11 and 5 convolutional layers, respectively, and a 3 × 3-kernel filter size. The supplied image must be a constant 224 × 224 pixels in size.

**Fig. 4:**
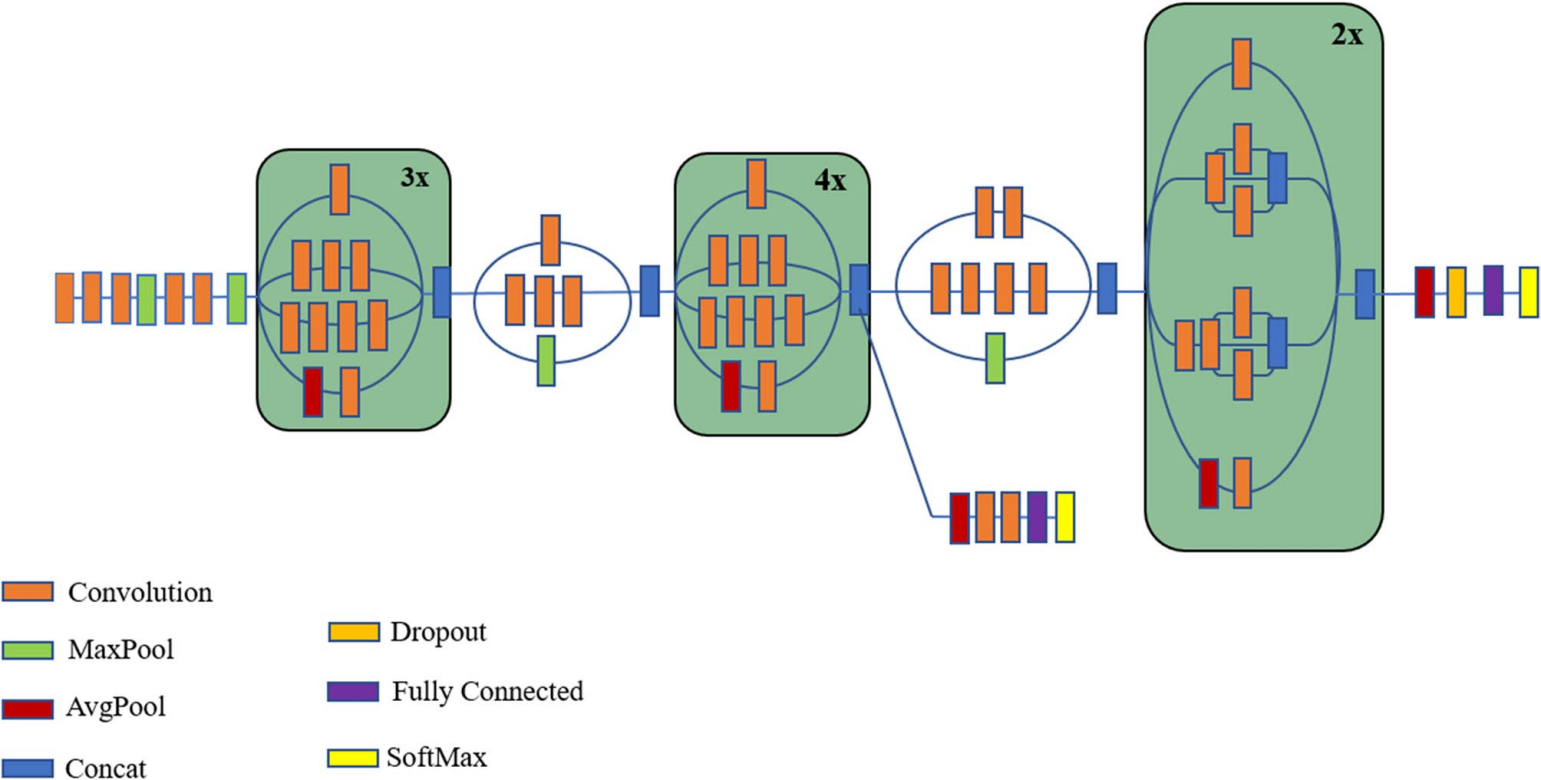
Architecture of Inception V3.

Following the pre-processing, the images were subjected to a convolutional layer using a 3 × 3 filter size. The input channel’s linear transformation has a filter size of 1 × 1. The length of the stride is set to 2 when max pooling is done using 2 × 2 sizes and the stride size is fixed at 1. Figure 4 shows the Inception V3 architecture for WMH analysis. In the stages that follow, each fully interconnected layer has an identical configuration and is made up of 4096 channels. After the last layer, which is the SoftMax activation layers, the activation function of RELU follows. It is possible to extract the CNN layer from either more feature maps or raw images. Here on this section of the network, the bulk of the user-specified parameters are saved. Both the overall number of kernels and their size are extremely important qualities. In particular, the following is the procedure for computing the convolution layer’s feature map in conjunction with a nonlinear activation function:

33 $$\:{\mathcal{v}}_{i,j,m}=Ma{x}_{m}K\times\:{P}_{i,j}+{\mathfrak{B}}_{m},0$$

Where, $\:{\mathcal{v}}_{i,j,m}$ indicates the activation factor of input image $\:m$, $\:i,j$ is the location of input image, $\:{P}_{i,j}$ denotes the location centered input patch, and $\:{\mathfrak{B}}_{m}$ is the bias value. Since the gradient stays high (which is comparable to 1) even when the neuron activates, the ReLU activation function is significant. To put it simply, the ReLU layer sets all negative activations to 0.0 and applies the function to every value in the source region. This layer improves the model’s and the network’s nonlinear properties without changing the receptive fields of the convolution layer.

34 $$\:{\mathcal{v}}_{i,j,m}=Max({\mathcal{S}}_{i,j,m},0)$$

The input to the activation function at the $\:{m}^{th}$ channel is represented by $\:{\mathcal{S}}_{i,j,m}$. The purpose of pooling layers is similar to that of convolution layers. For example, you can use them for max pooling, which finds the maximum value in a specific phishing region, or for average pooling, which finds the average value in a particular phishing space. The usual use of these procedures is to lower the network’s dimensionality.

35 $$\:{\mathcal{H}}_{i,j,m}={\left[{\sum\:}_{\left(a,b\right)\in\:{\tau\:}_{i,j}}{\left({\mathcal{v}}_{a,b,m}\right)}^{z}\right]}^{1/z}$$

The result of pooling at region $\:(i,j)$ in the feature map is represented by $\:{\mathcal{H}}_{i,j,m}$, and the feature value at location $\:(a,b)$ with the pooling region $\:{\tau\:}_{i,j}$ is $\:{\mathcal{v}}_{a,b,m}$. Helping to extract characteristics that are both lightweight and crisp is the primary goal of pooling. Also, it’s done to cut down on calculations and mistakes. With max-pooling, it’s easy to get rid of features like points and edges that aren’t really important. The most useful characteristic will be the area of the function map that the filter protects. After the max-pooling layer, a feature map containing the most important elements from the previous map will be produced. The following is the mathematical representation of this operation:

36 $$\:{h}_{i,j,m}=\beta\:\underset{(a,b)\in\:{\tau\:}_{i,j}}{\mathrm{max}}{\mathcal{v}}_{a,b,m}+(1-\beta\:)\frac{1}{\left|{\tau\:}_{i,j}\right|}\sum\:_{(a,b)\in\:{\tau\:}_{i,j}}{\mathcal{v}}_{a,b,m}$$

If the value of β is either 0 or 1, then the choice to use maximum pooling or average pooling is indicated. In order to enhance the network’s performance during classification, fully connected layers are utilized to flatten the results prior to classification. Identical to an MLP’s output layer. Dropout is a regularization strategy for neural networks that helps to decrease the learning of interdependent neurons. The term “dropout” is used to characterize units that exit a neural network, whether they are visible or not. The process of forgetting neurons, or units, selected at random from any set of neurons during the learning stage is called dropout.

37 $$\:\mathcal{y}=r\times\:\mathcal{v}\left({\omega\:}^{K}s\right)$$

38 $$\:s={[{s}_{1},{s}_{2}\dots\:{s}_{n}]}^{K}$$

The input of the fully linked layer is denoted by s, the weight matrix by ϋ, the size of the matrix by r, and the final output by y. In order to generate a probability distribution, the SoftMax algorithm takes an integer vector as input, normalizes it, and then uses the input integer’s exponential functions as coefficients. Here we see the mathematical representation of the non-local linearity distribution used by the values of units in a SoftMax category:

39 $$\:\mathcal{P}\left(x=z|y\right)=\frac{{e}^{{y}_{k}}}{\sum\:_{j}{e}^{{y}_{j}}}$$

Where, $\:y$ and $\:z$ are the probability values obtained from the SoftMax layer.

#### Particle Swarm Optimization (PSO) for training the neural network weights and hyperparameter tuning

This work explores the efficacy of PSO in enhancing the control parameters of proposed model within a mixed network environment. The network comprises both WMH and non WMH, represented by the model, and PSO train the parameters (See Algorithm 1). Specifically, the network features a closed loop with a merge lane, allowing MRI image to exit and re-enter the loop. This work proposes leveraging PSO within a deep learning framework for WMH segmentation. Utilizing the dataset containing WMH classes, augmentation techniques are employed to maintain balance and diversity. PSO is utilized to optimize and reduce the extracted features from the images. These optimized features are then inputted into a classification learner, where various classifiers are tested. In the final step, the acquired features undergo additional optimization using PSO, and the performance of different classifiers is assessed based on various metrics. In the PSO algorithm, each particle is influenced by two main factors: the position of the overall best solution found so far $\:{g}^{*}$ and its own best position in history $\:{x}_{i}^{*}$. However, particles also exhibit random movement tendencies. Whenever a particle discovers a better position than any it has previously encountered, PSO updates that position as its new personal best $\:{x}_{i}^{*}$. At any given iteration during the process, there exists a current best solution for each particle. The goal is to identify the global best solution among all the current individual best solutions until there is no further improvement in the WMH’s representation or after a specified number of iterations.

**Algorithm 1:**
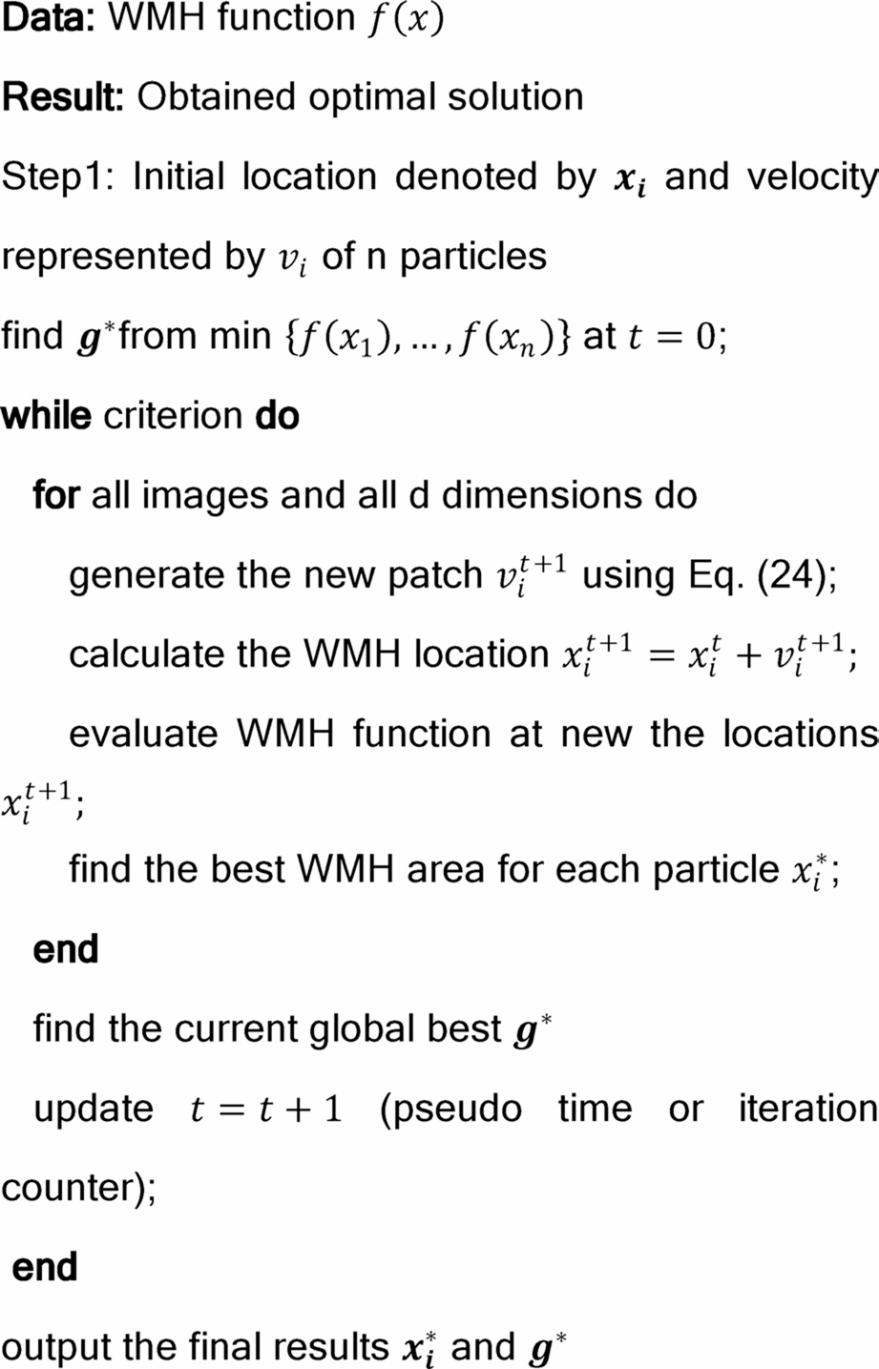
Particle Swarm Optimization.

Let $\:{x}_{i}$ and $\:{v}_{i}$ represent the position vector and velocity for a particle, respectively. The updated velocity vector is determined by the following formula.

40 $$\:{v}_{i}^{t+1}={v}_{i}^{t}+\alpha\:{ \varepsilon }_{1}\left[{g}^{*}-{x}_{i}^{t}\right]+\beta\:{ \varepsilon }_{2}[{x}_{i}^{*\left(t\right)}-{x}_{i}^{t}]$$

In this context, $\:{\epsilon\:}_{1}$and $\:{\epsilon\:}_{2}$denote two random vectors whose individual elements lie within the range [0, 1]. The coefficients $\:\alpha\:$ and $\:\beta\:$serve as learning or acceleration parameters, typically assigned values close to 2. For effective exploration of the search space—especially in multimodal optimization tasks—it is important that the initial positions of all particles are distributed as uniformly as possible. The initial velocity of each particle is generally set to zero, represented as $\:{v}_{i}^{(t=0)}=0$, after which particle positions are updated iteratively based on the defined velocity update rule.

41 $$\:{x}_{i}^{t+1}={x}_{i}^{t}+{v}_{i}^{t+1}\varDelta\:t$$

The time increment $\:\varDelta\:t$ is typically set to 1 for all implementations since PSO operates iteratively with a discrete integer time counter. Although $\:{v}_{i}$ can assume any values, it is commonly bounded within a specific range, such as [0, $\:{v}_{max}$]. Several variants extend the standard PSO algorithm, with one notable improvement being the use of an inertia function denoted as $\:{\theta\:}_{\left(t\right)}$, where $\:{v}_{i}^{t}$ is replaced by $\:\theta\:\left(t\right){v}_{i}^{t}$.

42 $$\:{v}_{i}^{t+1}=\theta\:{v}_{i}^{t}+\alpha\:{ \varepsilon }_{1}\left[{g}^{*}-{x}_{i}^{t}\right]+\beta\:{ \varepsilon }_{2}[{x}_{i}^{*\left(k\right)}-{x}_{i}^{t}]$$

When considering values between 0 and 1, θ represents a parameter. In the basic scenario, the inertia function can be simplified as a constant, usually around θ ≈ 0.5 to 0.9. This effectively introduces a virtual mass to steady the movement of the particles, thereby promoting quicker convergence of the algorithm.

### Proposed hybrid model

**Fig. 5:**
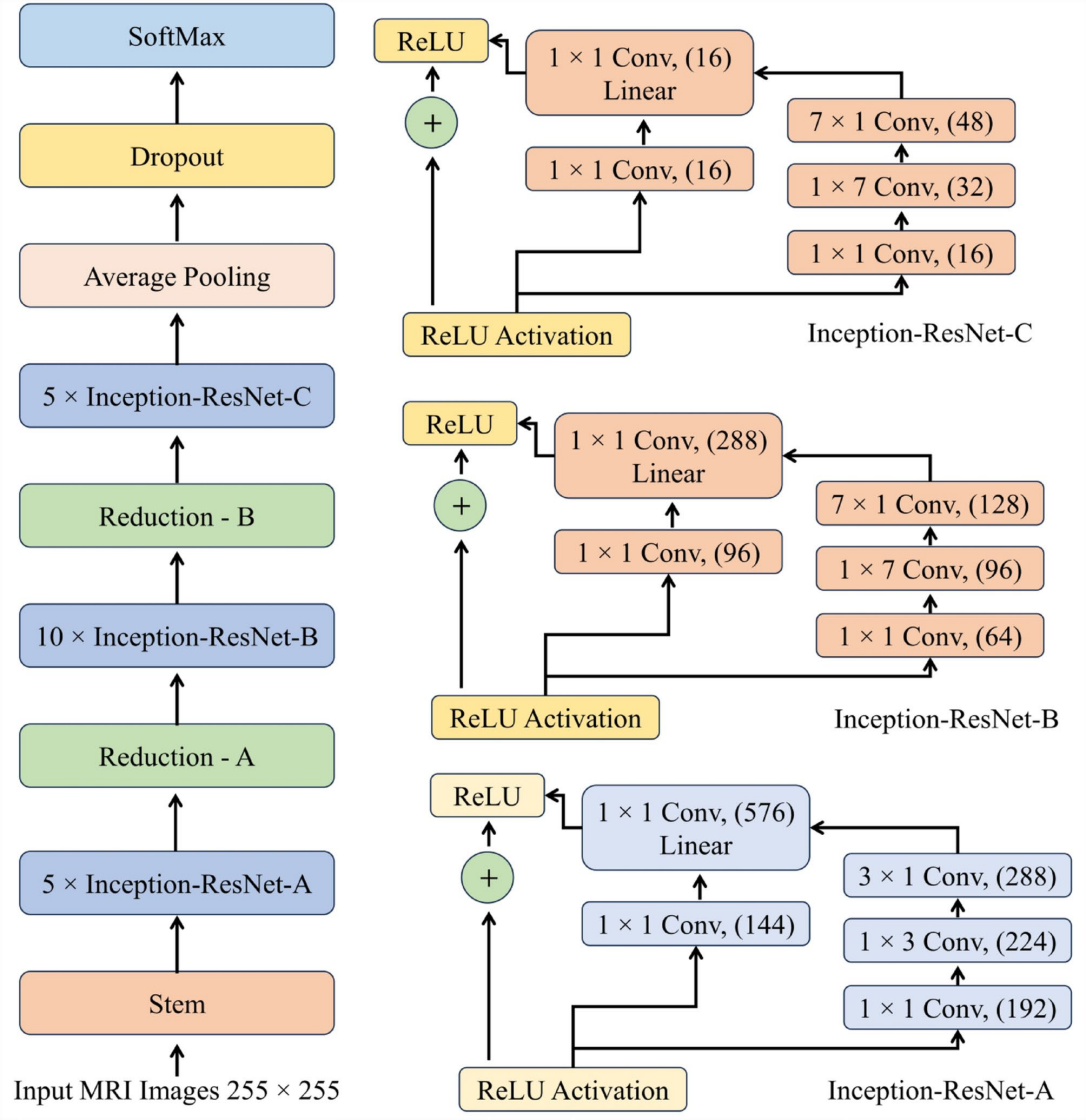
Proposed Inception-ResNet-v2 for brain WMH segmentation.

The proposed study aims to transform disease prediction and classification by creating a state-of-the-art hybrid model that combines the strengths of the Inception-v3 and ResNet50 architectures. Figure 5 shows the complex architecture of this hybrid method, which makes excellent use of the input images’ properties through its layered design. The utilization of both Inception-v3 and ResNet50 models serves a pivotal role in feature extraction from input images. Inception-v3, renowned for its exceptional performance in image recognition tasks, employs a sophisticated architecture that includes various inception modules, enabling the extraction of intricate features at different scales and levels of abstraction. On the other hand, ResNet50, with its revolutionary residual connections, effectively tackles the vanishing gradient problem and facilitates the training of deeper neural networks by ensuring smoother flow of gradients during backpropagation.

**Fig. 6:**
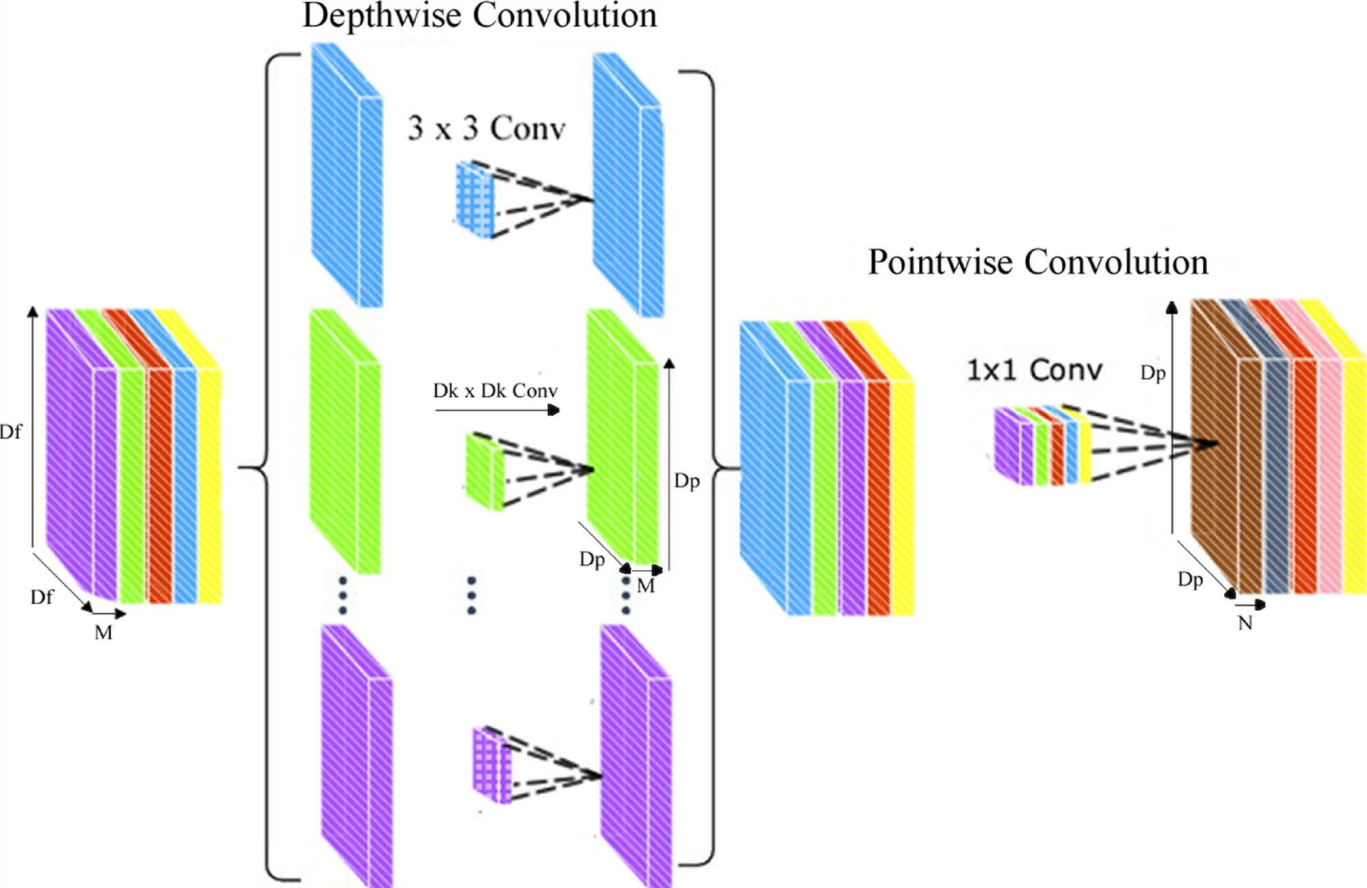
Depth wise convolution.

The core of the hybrid approach lies in the construction of a deep CNN (See Fig. 6) using features obtained from Inception-v3 and ResNet50 models. CNNs are renowned for their ability to automatically learn hierarchical representations of data, making them highly suitable for image classification tasks. By combining convolutional layers with additional components such as fully connected layers, pooling operations, and non-linear activation functions, the hybrid CNN architecture facilitates the extraction of discriminative features and the subsequent classification of input images into distinct disease categories. Furthermore, the training process of the hybrid model involves the utilization of backpropagation and convolutional filters. Backpropagation allows the model to iteratively adjust its parameters to minimize the prediction error, while convolutional filters specialize in tasks such as edge detection and classification. Although CNN filters are not highly customizable, their adaptability and ability to learn optimal filter values through training make them indispensable for image analysis tasks. ResNet50’s unique architecture plays a crucial role in simplifying the training of multilayer networks by minimizing training errors. Its deep architecture, coupled with residual connections, enables smoother gradient flow and facilitates the training of deeper models without encountering the vanishing gradient problem.

**Fig. 7:**
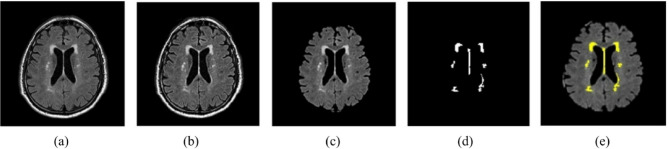
Represents the segmented WMH using proposed method. (**a**) original image, (**b**) enhanced image (**c**) skull stripping, (**d**) binary mask image, (**e**) Segmented output.

In contrast, while AlexNet pioneered the field of deep learning, its shallower architecture compared to ResNet50 may lead to architectural limitations and higher error rates. Additionally, ResNet50 offers a superior subspace value, minimizing the risk of feature overlap and enhancing the model’s discriminative power. The hybrid Inception-ResNet50-v3 model significantly enhances the accuracy and performance of WMH detection tasks (See Fig. 7), thanks to its robust architecture, efficient feature extraction capabilities, and advanced training mechanisms. By leveraging the strengths of both Inception-v3 and ResNet50 architectures, this hybrid model sets new benchmarks in disease prediction and classification, paving the way for innovative applications in the field of computer vision and healthcare. The Inception-ResNet50 model represents an evolution from the Inception V3 model, which drew inspiration from Microsoft’s ResNet paper on residual networks. This model enhances network depth while addressing issues like gradient disappearance and explosion, thereby improving overall network performance. By decomposing the convolution kernel, it increases network depth further, enhances computing power, and augments the nonlinearity of the network. This paper is built upon a fusion of the Inception-v3 and ResNet50 models. The model’s segmentation structure diagrams are illustrated in Fig. 7. Three identical modules, Inception-ResNet-A, Inception-ResNet-B, and Inception-ResNet-C, make up the feature extraction part of this hybrid model. Details like as the size of the convolution kernel, the number of channels, the step length, and any pooling or convolution processes are shown from left to right and top to bottom in the image, helping to define the rectangle. ‘V’ signifies valid (no filling), a value of 1 is used for marking, and ‘SAME’ means filling, the default. From the picture, readers can infer the shifts in the input and output dimensions. The concept of filters is depicted as tensor connections, where the number of channels after connection equals the sum of the preceding one. Despite the symmetric convolution kernels used in the Inception-ResNet-A module, asymmetric convolution kernels such 1 × 7, 7 × 1, 1 × 3, and 3 × 1 are utilized in the Inception-ResNet-B and Inception-ResNet-C modules (Algorithm 2).

**Algorithm 2:**
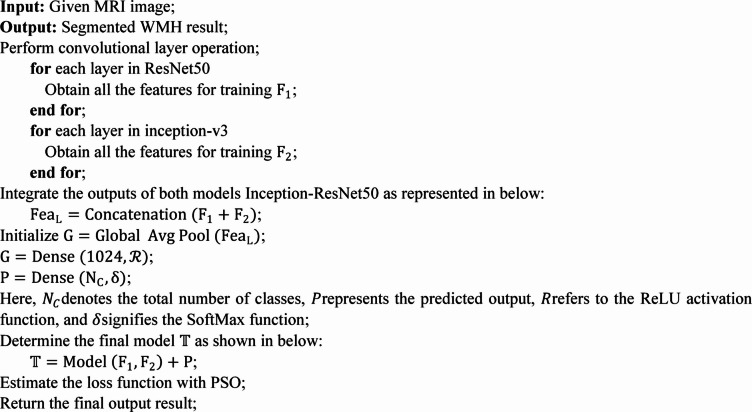
DOGHNN with PSO.

In addition to improving the network’s depth and nonlinearity, this method decreases computing time during parameter operations. The feature map is first processed using batch normalization and the ReLU activation function following a sequence of tensor connections and convolution operations. Finally, we get each module’s output. A fully connected layer and the SoftMax function are used for classification after all modules have been passed.

## Results

This section presents the results of extensive experiments that were carried out to assess the performance of the suggested algorithms and compare them to current approaches that are deemed state-of-the-art for handling WMH segmentation. Our new automated method, DOGHNN, was tested on a large dataset consisting of 900 individuals from various demographic backgrounds in order to isolate WMH from multimodal MRI scans. With its advanced labelling methodologies, the proposed methodology outperformed prior state-of-the-art methods in a quantitative evaluation of WMH segmentation of 2D MRI images using a wide range of imaging procedures and clinical diagnoses. This method for WMH segmentation was developed using the 2D thick-slice procedure, which is currently the standard for many scanners’ routine clinical acquisition software. It has the ability to identify and divide up a variety of pervasive vascular lesions in the brain. To provide a more thorough overview of WMH segmentation, the system can visually show the volumes and segmentation maps for each of the five categories that WMH can be categorized into (see to Fig. 5).

The suggested approach was successfully hick-validated using a dataset consisting of slice MRI images. Using the weighted Dice loss function, the proposed method was implemented using the Python DL toolbox. Images and marked areas were consistently down-sampled to a 0.5 pixel spacing on both the x and y axes to account for differences in scanner pixel spacing. On the z-axis, the pixel spacing did not alter. The picture volume was fed into 256 × 256 randomly chosen 2D patches during training. A 128-bit batch size, a learning rate of 0.001, and an NVIDIA GE Force 8 GB GPU were the training parameters. Testing and segmenting lesions from an unseen image took an average of under 15 s, in stark contrast to the 45 min required for training. The proposed strategy has numerous positive effects, one of which is the development of a competitive and practically relevant method for the automated segmentation of WMH. T1-weighted, T2-weighted, and FLAIR MRI sequences provide complementary information for WMH analysis. T1-weighted images offer clear anatomical structure and tissue contrast, T2-weighted images highlight fluid-sensitive regions, and FLAIR images suppress cerebrospinal fluid signals, making white matter hyperintensities more conspicuous. These modalities together enable reliable visualization and evaluation of WMH segmentation performance. Some examples of such results include the ability to detect WMH even when other brain lesions are present and the utilization of MRI images obtained using diverse imaging modalities.

**Fig. 8:**
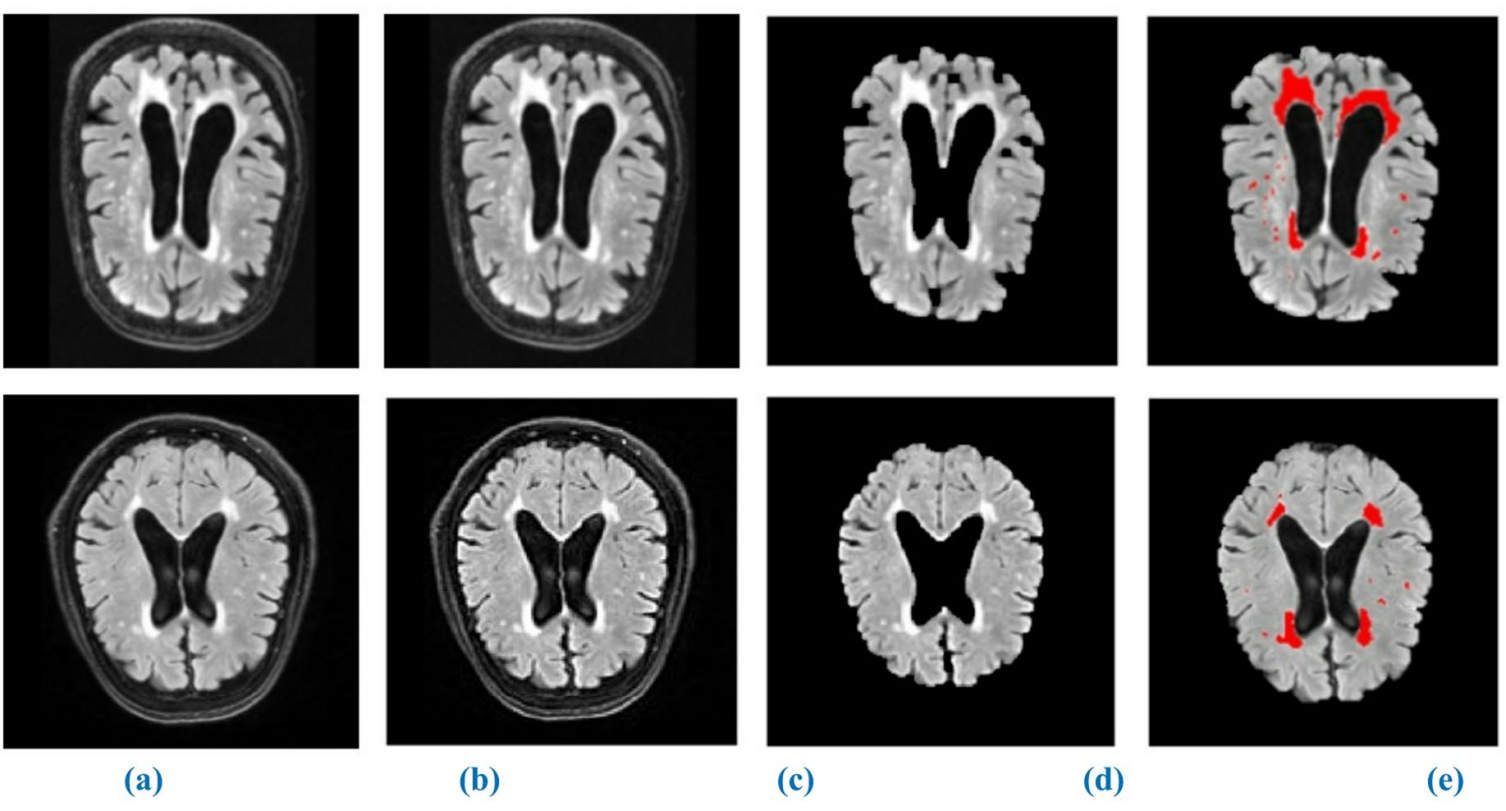
Segmentation of WMH on high-resolution T1-w MRI images. (**a**) original image, (**b**) enhanced image (**c**) skull stripping, (**d**) binary mask image, (**e**) Segmented output.

**Fig. 9:**
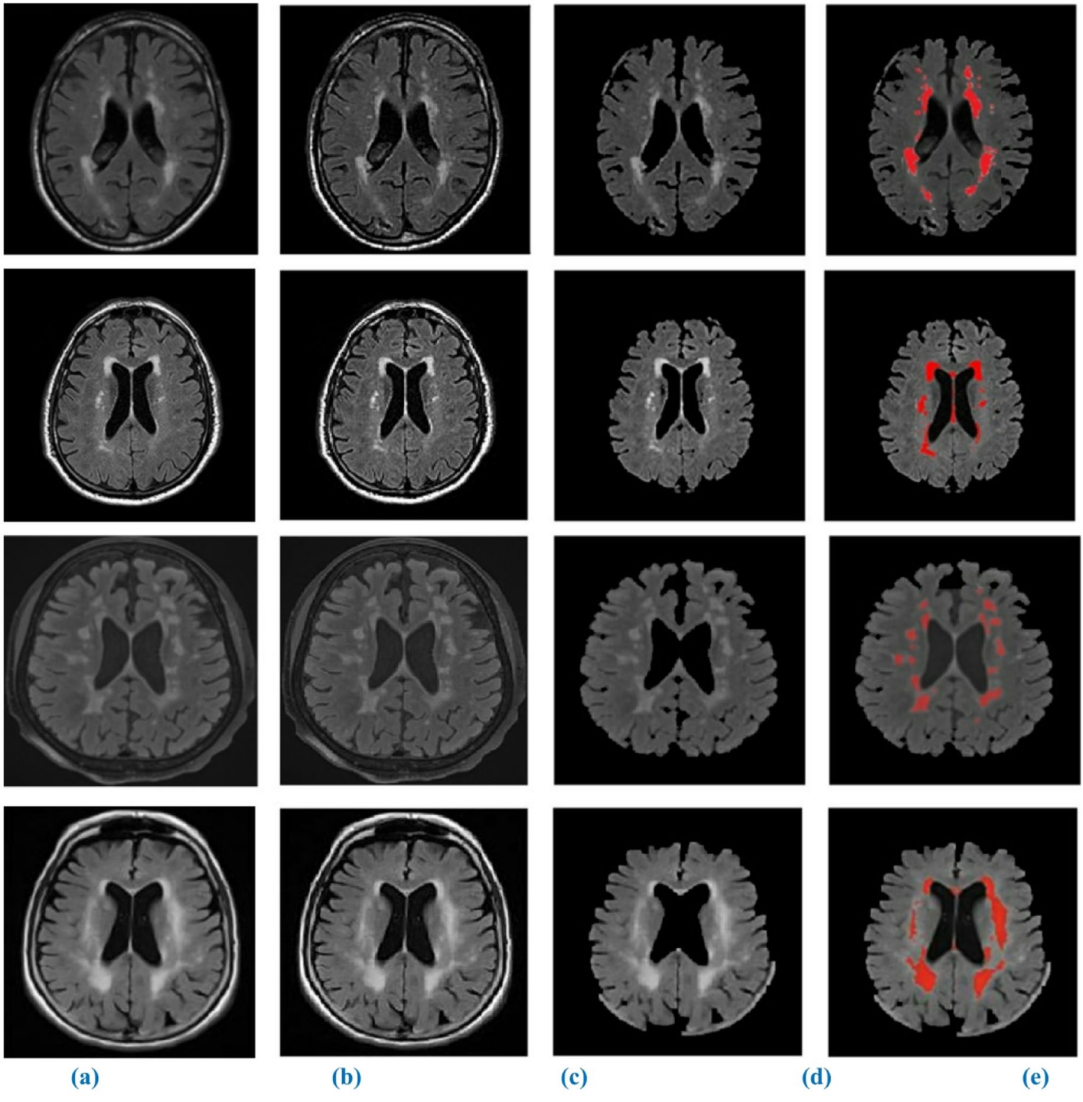
Segmentation of WMH on high-resolution T2-w MRI images. (**a**) original image, (**b**) enhanced image (**c**) skull stripping, (**d**) binary mask image, (**e**) Segmented output.

**Fig. 10:**
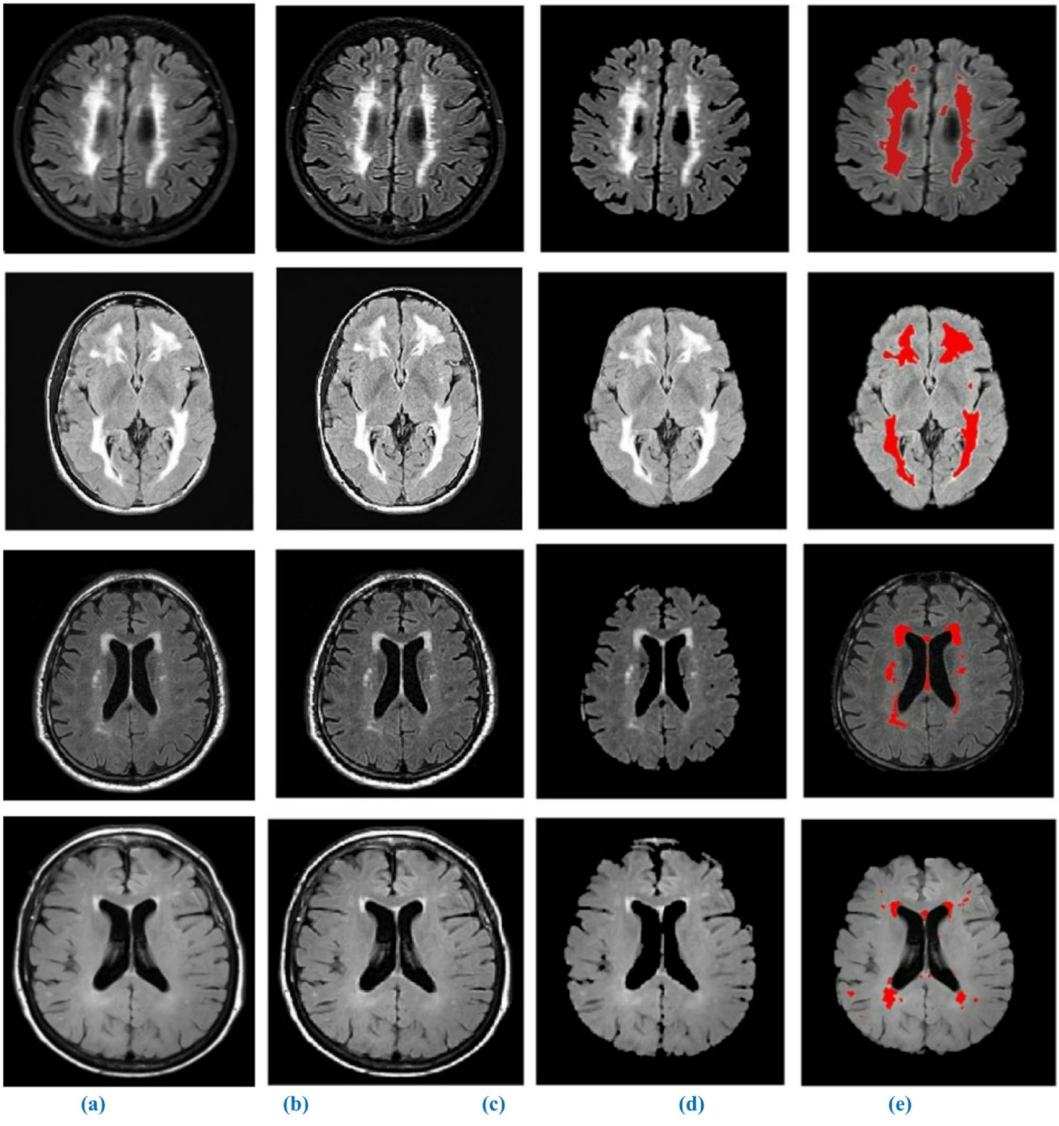
Segmentation of WMH on high-resolution FLAIR MRI images. (**a**) original image, (**b**) enhanced image (**c**) skull stripping, (**d**) binary mask image, (**e**) Segmented output.

Results of the WMH segmentation using our suggested model from T1-w, T2-w, and FLAIR MRI images are shown in Figs. 8 and 9, and 10 qualitatively. Since the suggested completely automated method could achieve delineation results that were on par with the “silver standard,” we also directly contrasted it with semi-automatic tools used for manual delineations carried out by a trained observer acting independently. We discovered that the fully automated strategy we proposed performed better than the semi-automatic one for WMH segmentation when we compared the two approaches. One of the strongest points of our study is the autonomous segmentation of WMH in many application settings, such as when WMH are present with other brain lesions and when using MRI data from several imaging operations. We presented a competitive method that is both clinically useable.

On the subject of WMH segmentation, it is worth mentioning that our suggested completely automatic method outperformed the human delineations performed by an expert observer utilizing semi-automatic tools (Table 5). Hence, we think our method is an acceptable option for WMH segmentation in real-world clinical settings.

### Quantitative evaluation criterion and rank method for WMH segmentation

**Fig. 11:**
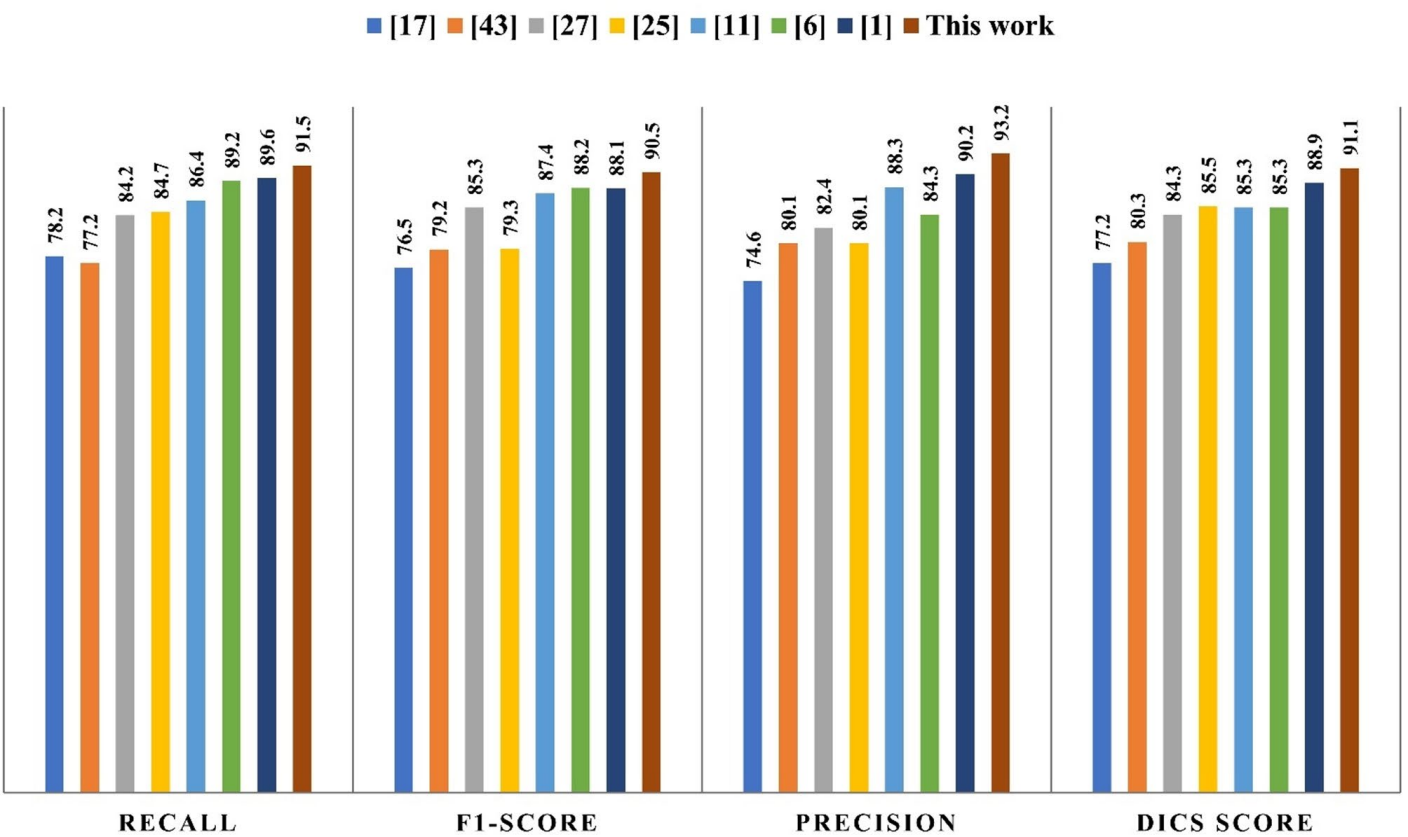
Quantitative comparison of differences between proposed algorithm and the other state-of-the-art algorithms for WMH segmentation.

**Fig. 12:**
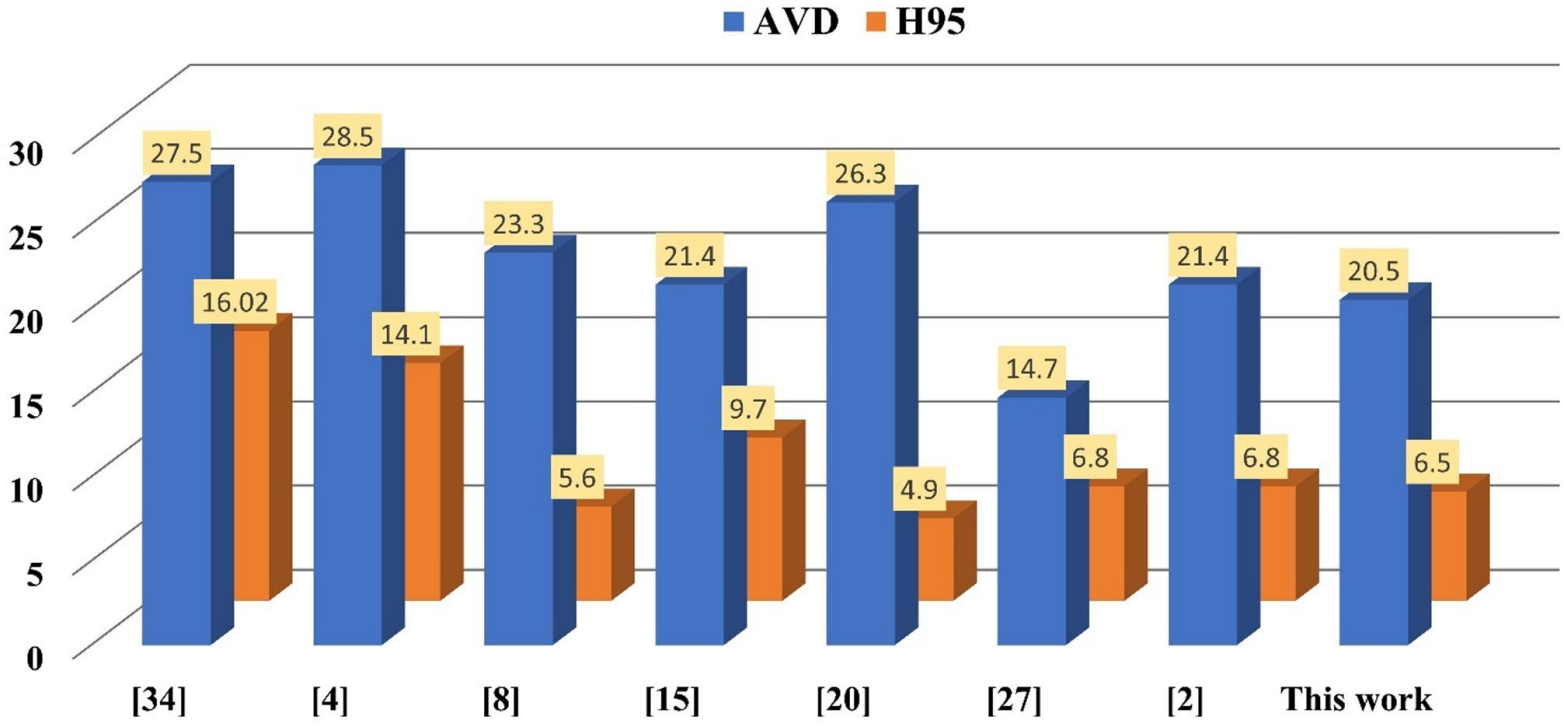
Represents the performance differences of AVD and H95 among the proposed and state-of-the-art algorithms for WMH segmentation.

In this study, the DOGHNN approach that was suggested is subjected to quantitative testing to evaluate its performance in terms of dice loss, f1-score, precision, and recall (See Figs. 11 and 12). Changing the training data is an integral part of quantitative research. To further prove the efficacy of the proposed method, the proposed DOGCNN trains on data from both the MACCAI challenging 2017 dataset and an internal dataset. G stands for the manual annotation, while P is the anticipated segmentation output. Each voxel is averaged for quantitative analytical metrics. Then, to focus in on WMH, we used weighted dice loss. Due to the reduction of WMH regions compared to background regions, there was an imbalance in the number of positive and negative samples. Attempts to segment medical imagery also ran across similar problems. To avoid training to become stuck in a loop of local minima, we used the Dice loss^1,6^(Eq. 43).

43 $$\:Dice\:loss=1-\frac{1}{C}\sum\:_{1}^{C}\left(\frac{2\sum\:_{i}^{N}{P}_{i}^{c}{g}_{i}^{c}}{\sum\:_{i}^{N}({P}_{i}^{c}{)}^{2}+\sum\:_{i}^{N}({g}_{i}^{c}{)}^{2}}\right)$$

where $\:{P}_{i}\in\:P$ is the output of the algorithm, $\:{g}_{i}\in\:G$ is the real-world value, and C is the total number of classes. Definite WMH and suspected WMH were defined previously. The Dice loss was adjusted since the definitive WMH was given greater consideration.

44 $$\:Dice\:loss=1-\frac{1}{C}\sum\:_{1}^{C}\left(w\left(x\right){)}^{c}\right(\frac{2\sum\:_{i}^{N}{P}_{i}^{c}{g}_{i}^{c}}{\sum\:_{i}^{N}({P}_{i}^{c}{)}^{2}+\sum\:_{i}^{N}({g}_{i}^{c}{)}^{2}})$$

Where, $\:(w\left(x\right){)}^{c}$ is the weight map.

The weighting factor was assigned a value of 2 for regions identified as confirmed WMH and defined as $\:1+f\left(dis\right(Dis\left)\right)$, where $\:dis\left(Dis\right)$represents the distance from a given point to the center of the nearest confirmed WMH region. The function $\:f\left(dis\right(Dis\left)\right)$was calculated such that its value lies within the range.

45 $$\:f\left(dis\left(Def\right)\right)=1-\frac{dis\left(Def\right))}{dis(Def{)}_{max}}$$

Dice Similarity Coefficient (DSC): DSC finds the proportion of space that is shared by G and P. A definition of it is

46 $$\:DSC=\frac{2\times\:|G\cap\:P|}{\left|G\right|+\left|P\right|}$$

Hausdorff Distance (HD): calculates the maximum variation between two sets by comparing the ground-truth (G) and automated segmentation (P) boundary distances. The $\:{K}^{th}$ ranked distance is used to disguise the outlier; a more robust version is made by substituting the $\:{95}^{th}$ percentile for the greatest distance.

47 $$\:h_{{95}} \left( {G,P} \right) = K_{{g \in G}}^{{th}} \begin{array}{*{20}c} {min} \\ {p\varepsilon P} \\ \end{array} \left\| {p - g} \right\|$$

Here, $\:G$ and $\:P$represent the boundary points of the manually annotated reference and the predicted segmentation, respectively. The term $\:95{K}_{(g\in\:G)}^{th}$refers to the Kth smallest Euclidean distance, determined such that $\:K/{N}_{g}=95\mathrm{\%}$. A smaller Hausdorff Distance (HD) value indicates a closer agreement between the predicted segmentation and the ground truth. The HD can be mathematically expressed as follows:

48 $$\:HD=\mathrm{m}\mathrm{a}\mathrm{x}\{{h}_{95}\left(G,P\right),\:{h}_{95}\left(P,G\right)\}$$

Recall: Recall detailed the rectified segmented section of the ground truth voxels as

49 $$\:recall=\frac{TP}{TP+FN}$$

Precision: Both the combined and separate sets of labelled WMH areas were used to determine the recall. The term “precision” describes the percentage of segmented voxels that are thought to be part of the ground truth regions.

50 $$\:precision=\frac{TP}{TP+FP}$$

The true positive (TP), true negative (FN), and false positive (FP) percentages are the relevant metrics in the aforementioned approaches. In addition to recall and accuracy at the voxel level, we also created the F1-score to measure performance. We used the same recall method as in equations^12,14,15^ for 3D linked component lesions, but we interpreted TP and FN as the percentage of successfully segmented lesions and the percentage of missing lesions, respectively.

***The F1-score*** was the harmonic mean of recall and precision and was calculated as:

51 $$\:F1=\frac{2*recall*precision}{recall+precision}$$

The purpose of developing lesion recall and F1-score was to improve voxel-level identification of memory and precision traits.

***Average Volume Difference*** (AVD) was defined as

52 $$\:AVD=\frac{|A-B|}{B}*100\%$$

with the segmented images denoted as A and the ground truth images as B. Model performance is evaluated using quantitative evaluation metrics. These metrics, which are based on previous work in the field of WMH segmentation^2,9,10,27^, comprise the SSIM^36,39^, the PSNR^40^, the MSE^41^, and the UQI^42^. Both the PSNR and the MSE measures are described as:

53 $$\:\boldsymbol{P}\boldsymbol{S}\boldsymbol{N}\boldsymbol{R}=10{log}_{10}\left(\frac{{k}_{max}^{2}}{MSE}\right)$$

54 $$\:\boldsymbol{M}\boldsymbol{S}\boldsymbol{E}=\frac{1}{m\times\:n}\sum\:_{i=1}^{m}\sum\:_{j=1}^{n}({k}_{ij}-({k}_{0}{)}_{ij}{)}^{2}$$

with $\:{K}_{max}$ = 255 for 8-bit images, and MSE standing for mean squared error. Image quality is directly proportional to the PSNR. For visual consistency evaluations within the range [0,1], structural similarity (SSIM) is a more resilient criterion because a score closer to 1 suggests more conservation of the structure. This statistic is based on people’s visual perception ability. The SSIM is calculated by utilizing two identically sized standard windows, A and B.

55 $$\:\boldsymbol{S}\boldsymbol{S}\boldsymbol{I}\boldsymbol{M}=\frac{\left(2{\mu\:}_{\omega\:1}{\mu\:}_{\omega\:2}+{c}_{1}\right)\:(2{\sigma\:}_{\omega\:1\omega\:2}+{c}_{2})}{\left(\mu\:{\omega\:}_{1}^{2}+\mu\:{\omega\:}_{2}^{2}+{c}_{1}\right)\:({\sigma\:}_{\omega\:1}^{2}+{\sigma\:}_{\omega\:2}^{2}+{c}_{2})}$$

Here, $\:{\mu\:}_{{\omega\:}_{i}}$and $\:{\sigma\:}_{{\omega\:}_{i}}^{2}$denote the mean and variance of the window $\:{\omega\:}_{i}$, respectively, while the covariance between two windows is represented as $\:{\sigma\:}_{{\omega\:}_{1}{\omega\:}_{2}}$. The constants $\:{c}_{1}$and $\:{c}_{2}$are stabilizing parameters introduced to maintain numerical stability during computation. To assess performance, the proposed approach was also evaluated against existing methods using the Universal Quality Index (UQI), a precursor to the Structural Similarity Index Measure (SSIM). The ground truth image is represented as $\:{I}_{gt}=\{{I}_{g{t}_{i}}{\hspace{0.17em}}\mid\:{\hspace{0.17em}}i=\mathrm{1,2},\dots\:,Z\}$, and the corresponding predicted image as $\:{I}_{pred}=\{{I}_{pre{d}_{i}}{\hspace{0.17em}}\mid\:{\hspace{0.17em}}i=\mathrm{1,2},\dots\:,Z\}$.

The average and standard deviation of window $\:{\omega\:}_{i}$ are represented by $\:{\mu\:}_{\omega\:i}$ and $\:{\sigma\:}_{\omega\:i}^{2}$, respectively. Covariance is represented as $\:{\sigma\:}_{\omega\:1\omega\:2}$, and numerical analysis makes use of the stabilizing factors $\:{c}_{1},{c}_{2}$ to guarantee stability.

**Table 5:** Quantitative evaluation for automatic WMH segmentation.

Method/teams	Dice score %	Precision%	Recall%	F1-score %	AVD$\:\downarrow\:$	H95$\:\downarrow\:$	PSNR	SSIM	UQI	MSE
Alamoudi et al.^4^	72.60	71.20	70.20	63.50	21.53	7.65	–	–	–	–
Lee et al.^3^	89.00	85.00	–	90.01	–	–	–	–	–	–
Bawil et al.^2^	80.00	–	90.00	86.00	18.3	6.20	–	–	–	–
Gibson et al.^10^	88.00	–	86.00	85.00	–	–	–	–	–	–
Eldianto et al.^16^	88.90	–	89.20	86.30	17.10	4.44	–	–	–	–
Zhang et al.^17^	89.00	88.00	–	85.00	6.70	×	–	–	–	–
Philps et al.^11^	83.30	85.80	88.50	–	13.70	6.28	–	–	–	–
Huang Y et al.^12^	88.00	90.10	91.00	–	–	–	–	–	–	–
Banu et al.^6^	85.30	91.70	91.20	83.73	–	–	–	–	–	–
Shan W et al.^24^	86.00	87.40	×	84.00	–	2.41	–	–	–	–
Umapathy et al.^25^	90.00	90.23	91.00	82.00	–	3.62	–	–	–	–
Pitkanen et al.^29^	87.00	90.10	90.00	88.00	–	×	–	–	–	–
Hong et al.^31^	90.60	–	89.91	83.40	–	17.47	–	–	–	–
Zhang et al.^14^	91.00	–	89.00	89.00	18.58	5.63	–	–	–	–
Pilli R et al.^15^	90.00	90.76	90.00	84.00	21.88	6.30	–	–	–	–
Rieu et al.^22^	–	–	–	–	–	–	28.33	0.80	0.98	54.11
Xu et al.^40^	–	–	–	–	–	–	38.63	0.75	0.92	48.21
Tai JZ S et al.^45^	91.93	89.76	89.23	88.87	–	–	–	–	–	–
Coenen et al.^13^	90.00	×	83.00	88.01	19.3	6.72	–	–	–	–
This work	91.1	93.2	91.5	90.5	20.5	6.5	40.44	0.89	0.90	36.23

**Fig. 13:**
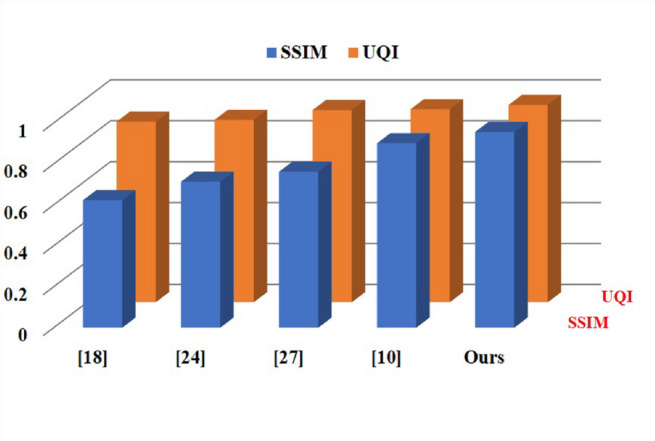
Quantitative comparison of SSIM and UQI for WMH segmentation.

**Fig. 14:**
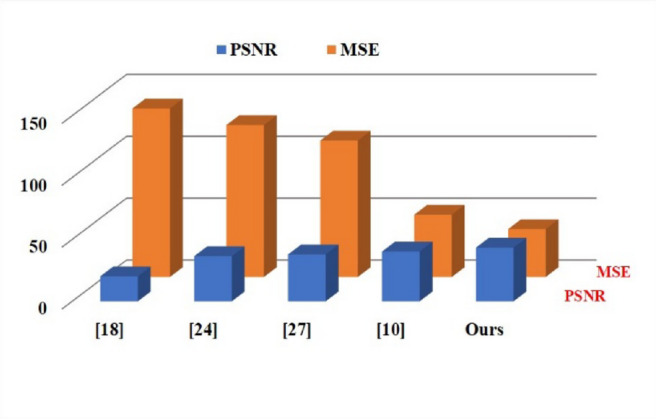
Quantitative comparison of PSNR and MSE of WMH segmentation.

The metric UQI, which was previously used to compare approaches to SSIM, was also used to compare our suggested methods to other methods. The qualitative comparison of metrics such as PSNR, SSIM, UQI, and MSE is illustrated in Figs. 13 and 14. Whereas the predicted image is denoted as $\:{I}_{pred}=\left\{{I}_{pred}|i=\mathrm{1,2},\dots\:,Z\right\}$ and the genuine image is represented as $\:{I}_{gt}$, the former is defined as $\:{I}_{gt}=\left\{{I}_{g{t}_{i}}|i=\mathrm{1,2},\dots\:,Z\right\}$. One definition of the UQI is:

56 $$\:\boldsymbol{U}\boldsymbol{Q}\boldsymbol{I}=\frac{{\sigma\:I}_{gt}{I}_{pred}}{{\sigma\:I}_{gt}{\sigma\:I}_{pred}}\cdot\:\frac{2\stackrel{-}{{I}_{gt}}\stackrel{-}{{I}_{pred}}}{(\stackrel{-}{{I}_{gt}}{)}^{2}+(\stackrel{-}{{I}_{pred}}{)}^{2}}\cdot\:\frac{2{\sigma\:I}_{gt}{\sigma\:I}_{pred}}{{{\sigma\:}^{2}I}_{gt}+{{\sigma\:}^{2}I}_{pred}}$$

The Universal Quality Index (UQI) operates within a dynamic range of $\:[-\mathrm{1,1}]$. The maximum value of 1 is achieved only when $\:{I}_{{\mathrm{pred}}_{i}}={I}_{{\mathrm{gt}}_{i}}$for all $\:i=\mathrm{1,2},\dots\:,Z$. Conversely, the minimum value of -1 occurs when $\:I_{{{\mathrm{pred}}_{i} }} = 2\overline{I} _{{{\mathrm{gt}}}} - I_{{{\mathrm{gt}}_{i} }}$for all $\:i$. The term $\:\frac{{\sigma\:}_{{I}_{\mathrm{gt}}{I}_{\mathrm{pred}}}}{{\sigma\:}_{{I}_{\mathrm{gt}}}{\sigma\:}_{{I}_{\mathrm{pred}}}}$, ranging from $\:[-\mathrm{1,1}]$, represents the correlation coefficient between the ground truth image $\:{I}_{\mathrm{gt}}$and the predicted image $\:{I}_{\mathrm{pred}}$, quantifying their linear relationship. The component $\:\frac{{2\overline{I} _{{{\mathrm{gt}}}} \overline{I} _{{{\mathrm{pred}}}} }}{{(\overline{I} _{{{\mathrm{gt}}}} )^{2} + (\overline{I} _{{{\mathrm{pred}}}} )^{2} }}$measures the similarity in mean intensity values between the two images, with a possible range of $\:\left[\mathrm{0,1}\right]$. This value equals 1 only when the mean intensities of both images are identical, i.e., $\:\overline{I} _{{{\mathrm{gt}}}} = \overline{I} _{{{\mathrm{pred}}}}$. The final term, $\:\frac{2{\sigma\:}_{{I}_{\mathrm{gt}}}{\sigma\:}_{{I}_{\mathrm{pred}}}}{{\sigma\:}_{{I}_{\mathrm{gt}}}^{2}+{\sigma\:}_{{I}_{\mathrm{pred}}}^{2}}$, indicates the contrast similarity between the ground truth and predicted images. Its value also lies within $\:[-\mathrm{1,1}]$, attaining the maximum of 1 only when the standard deviations of both images are equal ($\:{\sigma\:}_{{I}_{\mathrm{gt}}}={\sigma\:}_{{I}_{\mathrm{pred}}}$). The quality of the quantitative and qualitative WMH segmentation that was produced by partial convolution^18^ was inadequate. We found that the proposed strategy outperformed the alternatives. We further validated the generalization and robustness of our approaches by conducting other complementary measures. Finally, our WMH volume-Fazekas correlations were comparable to manual classification. The recommended approach was also more consistent with hand delineations than previous methods, especially for DWMH. This suggests that the suggested automatic technique can replace manual WMH segmentation in clinical practice and research. There are flaws in our study. WMHs and other disorders may coexist or merge; T2-FLAIR MR imaging may show stroke lesions. This study aimed to automatically segment WMHs, so we did not need to name other disorders. We unintentionally tagged the backdrop and other diseases the same when training the model. Due to the fact that stroke lesions can also be hyperintense, WMH segmentation results were falsely positive.

### Ablation experiment

To evaluate the contribution of each module in the proposed Deep Optimization-Guided Hybrid Neural Network (DOGHNN), a systematic ablation study was performed. While the complete DOGHNN integrates Inception-v3, ResNet-50, and Practical Swarm Optimization (PSO) to achieve state-of-the-art WMH segmentation, it is essential to understand the role of each component individually and how their combination leads to the observed performance improvements. This analysis provides insight into the necessity of the hybrid design and demonstrates how each module contributes to the overall robustness and accuracy of the framework (Table 6).

**Table 6:** Summarizes the results of the ablation experiments.

Model	Dice Score (%)	Precision (%)	Recall (%)	F1-Score (%)
Inception-v3 only	82.3	81.4	82.9	80.2
ResNet-50 only	84.6	82.0	82.0	80.7
PSO only (optimization layer)	80.5	81.5	85.5	81.5
DOGHNN (Inception-v3 + ResNet-50 + PSO)	91.1	93.2	91.5	90.5

The ablation results reveal distinct patterns in performance for each module. The Inception-v3 module, when evaluated alone, exhibits strong sensitivity to lesions of varying sizes due to its multi-scale feature extraction capability. This enables effective detection of small punctate WMHs but provides limited contextual understanding, which can result in less precise boundary delineation. The ResNet-50 module, in isolation, extracts deep contextual features that improve discrimination between lesions and surrounding tissue, particularly in regions with complex anatomy. However, it is less effective at capturing fine-grained lesion structures, which can reduce sensitivity to smaller lesions. The PSO module, evaluated independently, optimizes key parameters such as fusion weights and segmentation thresholds, improving overall segmentation quality, yet lacks the feature extraction capacity to achieve high accuracy on its own. The complete DOGHNN framework, integrating Inception-v3, ResNet-50, and PSO, achieves the highest performance across all metrics.

This outcome demonstrates that the combination of multi-scale feature extraction, deep contextual representation, and optimization-guided refinement produces synergistic improvements that surpass the performance of any individual module. The hybrid integration enables the model to simultaneously capture fine-grained lesion details and maintain robust global feature learning, resulting in improved Dice Score, Precision, Recall, and F1-Score. Overall, the ablation study confirms that each component contributes uniquely to the segmentation performance. The performance gains of DOGHNN are not attributable to a single module but emerge from the carefully designed interaction between Inception-v3, ResNet-50, and PSO. This analysis highlights the importance of component synergy in hybrid neural networks and provides a clear rationale for the proposed architecture, demonstrating that the integrated design is essential to achieving optimal segmentation accuracy and robustness in heterogeneous WMH datasets.

## Conclusion

The DOGHNN automatic white matter hyperintensities segmentation algorithm has been shown to be reliable and useful as an addition to current best practices for WMH patients. Automatic WMH segmentation from T1, T2, and FLAIR MRI images in MACCAI and in-house datasets is achieved by the method, and it takes a tolerable 45 min to process. In comparison to previous approaches, DOGHNN achieves better segmentation accuracy and spatial agreement when tested on a diverse sample of 900 individuals. The individuals were acquired from multiple MRI scanners and displayed a range of diagnoses and lesion loads. With optimization for automatic WMH segmentation, the suggested method appears to be quick, dependable, and user-friendly. The method’s pre-processing stage makes it versatile enough to improve image quality, which means it can handle low-resolution inputs with ease and yet produce good results. In comparison to all previous methods, the proposed DOGHNN achieved the highest possible accuracy (93.2%), recall (91.5%), dice score (91.1%), and f1-score (90.5%). Beyond WMH segmentation, the suggested approach has potential for other applications as well. The proposed method has the ability to be expanded to segment white matter, grey matter, and structural connections in brain MRI images by making use of the pre-processing stage and optimizing the methodology. This extension would make the procedure more efficient in computation and allow for a more thorough examination of brain structures. Improving diagnostic and treatment methods for neurological diseases is the ultimate goal of continuously improving and expanding the suggested method, which will allow for the unlocking of even greater potential for automated segmentation of brain MRI images.

## Data Availability

The datasets used and/or analysed during the current study available from: [https://doi.org/10.5281/zenodo.18604208](https:/doi.org/10.5281/zenodo.18604208) .

## Code availability

The custom code and implementation of the proposed model used to generate the results reported in this study are publicly available at Zenodo: 10.5281/zenodo.18604208. The archived repository contains the complete source code, model implementation, preprocessing scripts, and all necessary instructions to reproduce the experimental results presented in this paper.
